# Supplementary material for: Can phone surveys be representative in low- and middle-income countries? An application to Myanmar
Source: PLoS One. 2023 Dec 22;18(12):e0296292. doi: 10.1371/journal.pone.0296292 (PMC10745169; doi:10.1371/journal.pone.0296292)
Supplement: S1 File — (DOCX) [file pone.0296292.s001.docx]

# Supporting Information

S1 Table. Overview of the MHWS Questionnaire and Length of Interview Time

| **Module No.** | **Module** | **Number of questions** | **Average time taken (minutes) to administer** |
| --- | --- | --- | --- |
| 1 | Call information |  |  |
| 2 | Introduction, consent, and respondent information | 13 | 2 |
| 3 | Household composition | 14 | 1 |
| 4 | Recent migration | 27 | 1 |
| 5 | Household and agricultural assets | 18 | 2 |
| 6 | Respondent’s and other income earner’s income sources/employment | 6 | 1 |
| 7 | Household livelihoods and livelihood challenges | 23 | 5 |
| 8 | Non-Farm Business | 19 | 1 |
| 9 | Remittances and other Transfers | 16 | 1 |
| 10 | Livelihood Disruptions and Shocks | 15 | 4 |
| 11 | Coping and indebtedness | 18 | 4 |
| 12 | Diets and feeding practices | 20 | 6 |
| 13 | Food expenditures / consumption | 13 | 2 |
| 14 | Household Hunger Scale | 8 | 1 |
| 15 | Access to essential services | 7 | 2 |
| 16 | Closing | 4 | 1 |
|  | **Total** | **221** | **36** |

Source: Authors’

S2 Table. Overview of the MLCS 2017 Household Questionnaire and Length of Interview Time

| Module No. | Module Name | Number of questions | Average time taken to administer (in minutes) |
| --- | --- | --- | --- |
| 1 | Household member roster | 27 | 10 |
| 2 | Education, Literacy, Numeracy, and training | 24 | 9 |
| 3 | Health | 16 | 7 |
| 4 | Housing | 30 | 6 |
| 5a | Food Consumption in the Last 7 Days | 9 | 38 |
| 5b | Food Consumed away from Home | 5 | 1 |
| 5c | Non-Food consumption expenditure in the last 30 days | 3 | 8 |
| 5d | Non-food consumption expenditure in last 6 months | 6 | 9 |
| 6 | Household Durables | 5 | 10 |
| 7 | Labor and Employment | 60 | 11 |
| 8a | Parcel Roster | 23 | 2 |
| 8b | Harvest and agricultural labor | 9 | 6 |
| 8c | Inputs | 2 | 4 |
| 8d | Livestock | 22 | 3 |
| 8e | Aquaculture and Fishing | 12 | 1 |
| 8f | Agriculture, fishing machinery and equipment | 3 | 2 |
| 9 | Ownership of non-farm businesses | 20 | 3 |
| 10 | Finance | 4 | 4 |
| 11 | Shocks and coping strategies | 2 | 3 |
| 12 | Money senders in the last 12 months | 10 | 2 |
| 13 | Other income | 2 | 1 |
|  | Total | 294 | 140 |

Source: CSO, UNDP, and World Bank 2019b

**S3 Table. Population and household estimates and target sample sizes by State/Region**

| State/Region | Persons in conventional households - 2014^1^ | Number of households- 2014^1^ | Target sample size in Myanmar Household Welfare Survey (MHWS) |
| --- | --- | --- | --- |
| **Ayeyarwady** | 6,053,594 | 1,488,983 | 1,538 |
| **Bago** | 4,743,808 | 1,142,974 | 1,210 |
| **Chin** | 469,109 | 70,291 | 240 |
| **Kachin** | 1,370,748 | 269,365 | 408 |
| **Kayah** | 272,730^a^ | 44,294^a^ | 240 |
| **Kayin** | 1,454,264^a^ | 228,868^a^ | 374 |
| **Magway** | 3,786,538 | 919,777 | 974 |
| **Mandalay** | 5,843,424 | 1,323,191 | 1,533 |
| **Mon** | 1,949,821 | 422,612 | 511 |
| **Nay Pyi Taw** | 1,072,833 | 205,1606 | 288 |
| **Rakhine** | 2,034,148^a^ | 459,722^a^ | 522 |
| **Sagaing** | 5,076,326 | 824,766 | 1,324 |
| **Shan** | 5,500,933 | 1,169,569 | 1,448 |
| **Tanintharyi** | 1,352,283 | 201,259 | 350 |
| **Yangon** | 6,949,440 | 1,582,944 | 1,830 |
| **Total** | 47,929,999 | 10,877,832 | 12,790 |

Note: ^a^ This number likely only reflects the enumerated population of the Census survey rather than the total population. DoP (2015) note that an estimated 69,753 persons in Kayin State, 46,600 persons in Kachin State and 1,090,000 persons in Rakhine State were not counted during enumeration.

Source: ^1^Census 2014 (DoP, 2015), and Authors.

**S4 Table. Respondent characteristic targets for each state and region, in percentage of respondents**

| State/ Region | Gender (female) | Location (rural) | Education level (low) | Livelihood (farming) |
| --- | --- | --- | --- | --- |
| **Ayeyarwady** | 50 | 86 | 54 | 47 |
| **Bago** | 50 | 78 | 52 | 46 |
| **Chin** | 50 | 79 | 50 | 66 |
| **Kachin** | 50 | 64 | 42 | 43 |
| **Kayah** | 50 | 75 | 47 | 60 |
| **Kayin** | 50 | 78 | 57 | 45 |
| **Magway** | 50 | 85 | 56 | 52 |
| **Mandalay** | 50 | 65 | 49 | 41 |
| **Mon** | 50 | 72 | 52 | 33 |
| **Nay Pyi Taw** | 50 | 68 | 43 | 30 |
| **Rakhine** | 50 | 83 | 58 | 46 |
| **Sagaing** | 50 | 83 | 54 | 63 |
| **Shan** | 50 | 76 | 60 | 73 |
| **Tanintharyi** | 50 | 76 | 50 | 40 |
| **Yangon** | 50 | 30 | 31 | 13 |

Source: Authors.

**S5 Table. Overview of target and achieved number of respondents, in total and by selected target gaps**

| State/ Region | Total households surveyed | | | Rural location criterion | | | Low level education criterion | | | Farming household criterion | | |
| --- | --- | --- | --- | --- | --- | --- | --- | --- | --- | --- | --- | --- |
|  | Target sample size | Actual sample size | Gap from target (%) | Target sample size | Actual sample size | Gap from target (%) | Target sample size | Actual sample size | Gap from target (%) | Target sample size | Actual sample size | **Gap from target (%)** |
|  | **Round 1** | | | | | | | | | | | |
| **Ayeyarwady** | 1,538 | 1,538 | 0.0% | 1,323 | 1,322 | -0.1% | 831 | 822 | -1.1% | 721 | 726 | 0.7% |
| **Bago** | 1,210 | 1,169 | -3.4% | 944 | 921 | -2.4% | 629 | 571 | -9.2% | 558 | 577 | 3.4% |
| **Chin** | 240 | 159 | -33.8% | 190 | 108 | -43.2% | 120 | 29 | -75.8% | 158 | 96 | -39.2% |
| **Kachin** | 408 | 385 | -5.6% | 261 | 229 | -12.3% | 171 | 149 | -12.9% | 175 | 157 | -10.3% |
| **Kayah** | 240 | 132 | -45.0% | 180 | 71 | -60.6% | 113 | 26 | -77.0% | 144 | 81 | -43.8% |
| **Kayin** | 374 | 354 | -5.3% | 292 | 276 | -5.5% | 213 | 194 | -8.9% | 170 | 175 | 2.9% |
| **Magway** | 974 | 963 | -1.1% | 828 | 822 | -0.7% | 545 | 531 | -2.6% | 502 | 505 | 0.6% |
| **Mandalay** | 1,533 | 1,483 | -3.3% | 996 | 1,024 | 2.8% | 751 | 662 | -11.9% | 624 | 633 | 1.4% |
| **Mon** | 511 | 480 | -6.1% | 368 | 324 | -12.0% | 266 | 225 | -15.4% | 168 | 169 | 0.6% |
| **Nay Pyi Taw** | 288 | 289 | 0.3% | 196 | 206 | 5.1% | 124 | 123 | -0.8% | 87 | 89 | 2.3% |
| **Rakhine** | 522 | 526 | 0.8% | 433 | 441 | 1.8% | 303 | 296 | -2.3% | 241 | 245 | 1.7% |
| **Sagaing** | 1,324 | 1,312 | -0.9% | 1,099 | 1,084 | -1.4% | 715 | 708 | -1.0% | 831 | 835 | 0.5% |
| **Shan** | 1,448 | 1,156 | -20.2% | 1,100 | 851 | -22.6% | 869 | 597 | -31.3% | 1,058 | 811 | -23.3% |
| **Tanintharyi** | 350 | 328 | -6.3% | 266 | 231 | -13.2% | 175 | 133 | -24.0% | 140 | 125 | -10.7% |
| **Yangon** | 1,830 | 1,826 | -0.2% | 549 | 581 | 5.8% | 567 | 554 | -2.3% | 232 | 241 | 3.9% |
| **Total** | 12,790 | 12,100 | -5.4% | 9,025 | 8,491 | -5.9% | 6,392 | 5,620 | -12.1% | 5,809 | 5,465 | -5.9% |
|  | **Round 4** | | | | | | | | | | | |
| **Ayeyarwady** | 1,538 | 1542 | 0.3% | 1,323 | 1335 | 0.9% | 831 | 827 | -0.5% | 721 | 729 | 1.1% |
| **Bago** | 1,210 | 1210 | 0.0% | 944 | 955 | 1.2% | 629 | 630 | 0.2% | 558 | 571 | 2.3% |
| **Chin** | 240 | 281 | 14.6% | 190 | 195 | 2.6% | 120 | 105 | -14.2% | 158 | 179 | 11.7% |
| **Kachin** | 408 | 409 | 0.2% | 261 | 262 | 0.4% | 171 | 172 | 0.7% | 175 | 180 | 2.8% |
| **Kayah** | 240 | 258 | 7.0% | 180 | 175 | -2.9% | 113 | 119 | 5.0% | 144 | 148 | 2.8% |
| **Kayin** | 374 | 393 | 4.8% | 292 | 320 | 8.8% | 213 | 219 | 2.7% | 170 | 198 | 14.2% |
| **Magway** | 974 | 974 | 0.0% | 828 | 831 | 0.4% | 545 | 545 | 0.1% | 502 | 513 | 2.2% |
| **Mandalay** | 1,533 | 1533 | 0.0% | 996 | 1013 | 1.7% | 751 | 750 | -0.2% | 624 | 610 | -2.3% |
| **Mon** | 511 | 538 | 5.0% | 368 | 402 | 8.5% | 266 | 275 | 3.2% | 168 | 210 | 19.9% |
| **Nay Pyi Taw** | 288 | 288 | 0.0% | 196 | 199 | 1.5% | 124 | 122 | -1.5% | 87 | 88 | 1.3% |
| **Rakhine** | 522 | 522 | 0.0% | 433 | 432 | -0.2% | 303 | 266 | -13.8% | 241 | 246 | 2.0% |
| **Sagaing** | 1,324 | 1324 | 0.0% | 1,099 | 1097 | -0.2% | 715 | 710 | -0.8% | 831 | 837 | 0.7% |
| **Shan** | 1,448 | 1447 | -0.1% | 1,100 | 1099 | -0.1% | 869 | 838 | -3.7% | 1,058 | 1035 | -2.3% |
| **Tanintharyi** | 350 | 370 | 5.4% | 266 | 296 | 10.1% | 175 | 179 | 2.3% | 140 | 162 | 13.6% |
| **Yangon** | 1,830 | 1835 | 0.3% | 549 | 612 | 10.3% | 567 | 558 | -1.6% | 232 | 233 | 0.4% |
| **Total** | 12,790 | 12924 | 1.0% | 9,025 | 9223 | 2.1% | 6,392 | 6307 | -1.3% | 5,809 | 5945 | 2.3% |

Source: Authors.

**S6 Table. Township characteristics of townships not enumerated in MHWS**

| **State** | Township | **Population size ^a^** | **Number of households ^a^** | **Sample target ^b^** | **Round not enumerated** | **Enumerated in MLCS?** |
| --- | --- | --- | --- | --- | --- | --- |
| **Shan (North)** | Pangsang | 88,732 | 16,457 | 26 | R1,R2,R3,R4 | No |
| **Shan (North)** | Narphan | 114,724 | 16,474 | 29 | R1,R2,R3,R4 | No |
| **Shan (North)** | Pangwaun | 96,940 | 13,969 | 24 | R1,R2,R3,R4 | No |
| **Shan (North)** | Mongmao | 69,364 | 10,445 | 18 | R1,R2,R3,R4 | No |
| **Shan (North)** | Hopang | 59,438 | 11,216 | 15 | R1,R2,R3,R4 | Yes |
| **Shan (North)** | Matman | 19,050 | 3,318 | 5 | R1,R2,R3,R4 | No |
| **Shan (North)** | Konkyan | 59,565 | 9,665 | 15 | R1,R2,R3,R4 | No |
| **Shan (North)** | Mongyai | 56,768 | 13,328 | 15 | R1,R2,R3,R4 | Yes |
| **Shan (North)** | Namtu | 50,423 | 11,641 | 13 | R3,R4 | Yes |
| **Shan (North)** | Manton | 37,254 | 12,521 | 10 | R4 | Yes |
| **Shan (South)** | Langkho | 38,344 | 9,548 | 10 | R1,R2,R3 | Yes |
| **Shan (South)** | Mongpan | 23,503 | 5,421 | 6 | R1,R2,R3,R4 | No |
| **Shan (South)** | Kyethi | 70,623 | 14712 | 18 | R2,R3,R4 | Yes |
| **Shan (South)** | Laihka | 48,831 | 8,790 | 12 | R3,R4 | Yes |
| **Shan (South)** | Mawkmai | 33,810 | 7,194 | 8 | R3,R4 | Yes |
| **Shan (East)** | Mongping | 65,886 | 13,299 | 17 | R1,R2,R3,R4 | No |
| **Shan (East)** | Monghpyak | 28,235 | 6,155 | 8 | R1,R2,R3,R4 | Yes |
| **Shan (East)** | Mongyawng | 75,413 | 17,196 | 20 | R1 R3,R4 | No |
| **Kachin** | Injangyang | 1,420 | 285 | 0 | R1,R2,R3,R4 | No |
| **Kachin** | Tsawlaw | 6,499 | 1,073 | 2 | R1,R2,R3,R4 | No |
| **Kachin** | Sumprabum | 2,405 | 479 | 1 | R1,R2,R3,R4 | No |
| **Kachin** | Machanbaw | 8,353 | 1,719 | 2 | R1,R2,R3,R4 | Yes |
| **Kachin** | Nawngmun | 7,025 | 1,212 | 2 | R1,R2,R3,R4 | No |
| **Kachin** | Khaunglanhpu | 11,635 | 1,711 | 3 | R1,R2,R3,R4 | No |
| **Yangon** | Cocokyun | 1,172 | 351 | 0 | R1,R2,R3,R4 | No |
| **Sagaing** | Wuntho | 69,442 | 14,205 | 18 | R2, R4 | Yes |
| **Sagaing** | Pinlebu | 109,289 | 21,900 | 28 | R4 | Yes |
| **Kayah** | Mese | 5,608 | 1,302 | 5 | R4 | Yes |
| **Total not enumerated (nationwide) ^a^** | | 1,221,407 | 236,038 | 320 |  |  |
| **Total (nationwide) ^a^** | | 51,144,607 | 11,162,510 | 12,790 |  |  |
| **Share of total not enumerated ^a^** | | 2.38% | 2.11% | 2.50% |  |  |
| **Total not enumerated (target) ^c^** | | 773,359 | 164,159 | 101 |  |  |
| **Total (target) ^c^** |  | 50,696,359 | 11,090,631 | 12,673 |  |  |
| **Share of target not enumerated ^c^** | | 1.53% | 1.48% | 0.80% |  |  |

Notes: ^a^ Numbers based on Census 2014, population in conventional households only. ^b^These are sample targets proportional to population size. SAZ=Special Administered Zone.  ^c^ Total number of persons in conventional households and households based on ICS 2019, this excludes townships from Wa SAZ. These numbers are provided for round 4, where we enumerated the fewest townships.

Source: DoP (2015), DoP and UNFPA (2020), Authors

**S7 Table. Monthly average of violent events and experience of any violence during survey period, at township level and relative to population size**

|  | **# conflicts per 10,000 households** | **% townships with conflict** | ***# townships*** |
| --- | --- | --- | --- |
| **Round 1** |  |  |  |
| **No respondents (0)** | 2.09 | 5 | 20 |
| **Underrepresented (≤7)** | 1.19 | 74 | 31 |
| **Normal representation (7-14)** | 0.81 | 78 | 234 |
| **Overrepresented (14-22)** | 1.57 | 69 | 36 |
| **Largely overrepresented (>22)** | 5.93 | 100 | 8 |
| **Round 2** |  |  |  |
| **No respondents (0)** | 3.40 | 19 | 21 |
| **Underrepresented (≤7)** | 1.30 | 70 | 53 |
| **Normal representation (7-14)** | 0.82 | 76 | 194 |
| **Overrepresented (14-22)** | 1.42 | 83 | 47 |
| **Largely overrepresented (>22)** | 2.71 | 86 | 14 |
| **Round 3** |  |  |  |
| **No respondents (0)** | 0.15 | 13 | 24 |
| **Underrepresented (≤7)** | 1.12 | 50 | 42 |
| **Normal representation (7-14)** | 0.85 | 63 | 208 |
| **Overrepresented (14-22)** | 1.79 | 80 | 45 |
| **Largely overrepresented (>22)** | 3.54 | 80 | 10 |
| **Round 4** |  |  |  |
| **No respondents (0)** | 0.45 | 33 | 27 |
| **Underrepresented (≤7)** | 1.18 | 52 | 31 |
| **Normal representation (7-14)** | 0.69 | 71 | 210 |
| **Overrepresented (14-22)** | 0.95 | 80 | 45 |
| **Largely overrepresented (>22)** | 1.87 | 69 | 16 |

Source: the authors’ estimates from MHWS, MLCS and ACLED (number of battles, explosions, and violent events)

**S8 Table. Comparison of MHWS unweighted and weighted household composition and household wealth characteristics**

|  | **National** | | **Urban** | | **Rural** | |
| --- | --- | --- | --- | --- | --- | --- |
|  | **No weights** | **Weighted** | **No weights** | **Weighted** | **No weights** | **Weighted** |
| **child <5y** | 24% | 24% | 23% | 22% | 25% | 24% |
| **child 5-14y** | 48% | 48% | 44% | 44% | 49% | 50% |
| **adult 15-64y** | 100% | 100% | 100% | 100% | 100% | 99% |
| **senior >= 65y** | 22% | 22% | 21% | 21% | 23% | 23% |
| **# children <5y** | 0.27 | 0.27 | 0.26 | 0.25 | 0.28 | 0.28 |
| **# children 5-14y** | 0.69 | 0.70 | 0.63 | 0.62 | 0.72 | 0.72 |
| **# adults 15-64y** | 3.15 | 3.06 | 3.18 | 3.12 | 3.13 | 3.04 |
| **# seniors >= 65y** | 0.29 | 0.28 | 0.26 | 0.25 | 0.30 | 0.29 |
| **Women adults only ^1^** | 6% | 9% | 7% | 10% | 6% | 9% |
| **Agricultural land owned** |  |  |  |  |  |  |
| **0 acre** | 53% | 63% | 83% | 90% | 41% | 53% |
| **0-2acre** | 12% | 10% | 5% | 4% | 14% | 12% |
| **2-4 acre** | 9% | 8% | 4% | 2% | 12% | 10% |
| **4-7.5 acre** | 11% | 9% | 4% | 2% | 14% | 11% |
| **>7.5 acre** | 15% | 10% | 4% | 2% | 20% | 13% |
| **Type of dwelling** |  |  |  |  |  |  |
| **Wood/bamboo house** | 66% | 67% | 53% | 48% | 71% | 74% |
| **Semi-pucca house** | 15% | 14% | 18% | 18% | 14% | 12% |
| **Bungalow** | 13% | 12% | 17% | 16% | 11% | 10% |
| **Apartment** | 4% | 5% | 11% | 17% | 1% | 1% |
| **Hut (2-3y)** | 2% | 2% | 1% | 1% | 2% | 2% |
| **Hut (1y)** | 1% | 1% | 0% | 0% | 1% | 1% |
| **Tenure status (dwelling)** |  |  |  |  |  |  |
| **Owned/free** | 91% | 90% | 77% | 74% | 97% | 96% |
| **Rented** | 9% | 9% | 22% | 25% | 3% | 3% |
| **Squatter** | 0% | 0% | 1% | 1% | 0% | 0% |
| **Camp, shelter** | 0% | 0% | 1% | 0% | 0% | 0% |
| **Improved floor** | 75% | 73% | 81% | 81% | 72% | 69% |
| **Improved source of drinking water** | 80% | 79% | 92% | 93% | 75% | 74% |

Note: ^1^ These are households with women adults (aged >14 years old) but without male adults (aged >14 years) in the household.

Source: the authors’ estimates from MHWS.

**S9 Table. Comparison of MHWS unweighted and weighted respondent-level descriptive characteristics, in percentage**

|  | **National** | | **Urban** | | **Rural** | |
| --- | --- | --- | --- | --- | --- | --- |
|  | **No weights** | **Weighted** | **No weights** | **Weighted** | **No weights** | **Weighted** |
| **Low education level** | 46 | 58 | 27 | 36 | 54 | 67 |
| **junior: 18-24** | 17 | 18 | 20 | 22 | 15 | 17 |
| **middle: 25-49y** | 62 | 60 | 64 | 62 | 61 | 59 |
| **senior: 50-74y** | 21 | 22 | 16 | 16 | 23 | 24 |
| **head** | 39 | 34 | 37 | 30 | 40 | 35 |
| **spouse** | 24 | 23 | 22 | 21 | 24 | 24 |
| **child** | 28 | 33 | 31 | 37 | 27 | 31 |
| **other** | 4 | 5 | 6 | 8 | 4 | 4 |
| **Female** | 50 | 53 | 50 | 53 | 51 | 53 |

Source: the authors’ estimates from MHWS.

**S10 Table. Attrition of round 1 and round 2 households in the sample**

|  | **R1** | **R2** | **R3** | **R4** |
| --- | --- | --- | --- | --- |
| Round 1 households remaining in sample |  | 7,799 | 6,567 | 5,172 |
| Round 1 attrition (share of round 1 households no longer interviewed in the respective following round) |  | 36% | 46% | 60% |
| Round 2 households remaining in sample |  |  | 3,063 | 2,358 |
| Round 2 attrition (share of round 2 households no longer interviewed in the respective following round) |  |  | 21% | 42% |
| Round 3 households remaining in sample |  |  |  | 1,670 |
| Round 3 attrition (share of round 3 households no longer interviewed in the respective following round) |  |  |  | 29% |
| Sample size per round | 12,100 | 12,142 | 12,128 | 12,924 |

Source: the authors’ calculations from MHWS

**S11 Table. Number of observations per round**

|  | **Freq.** | **Percent** |
| --- | --- | --- |
| R1, R2, R3 and R4 household | 19,324 | 39.2 |
| R1, R2, R3 household | 3,480 | 7.1 |
| R1, R3, R4 household | 1,023 | 2.1 |
| R2, R3, R4 household | 7,074 | 14.4 |
| R1, R2 household | 3,616 | 7.3 |
| R1, R3 household | 470 | 1.0 |
| R2, R3 household | 1,410 | 2.9 |
| R3, R4 household | 3,340 | 6.8 |
| R1 only | 3,725 | 7.5 |
| R2 only | 1,280 | 2.6 |
| R3 only | 828 | 1.6 |
| R4 only | 3,724 | 7.5 |
| Total | 49,294 | 100 |

Source: the authors’ calculations from MHWS


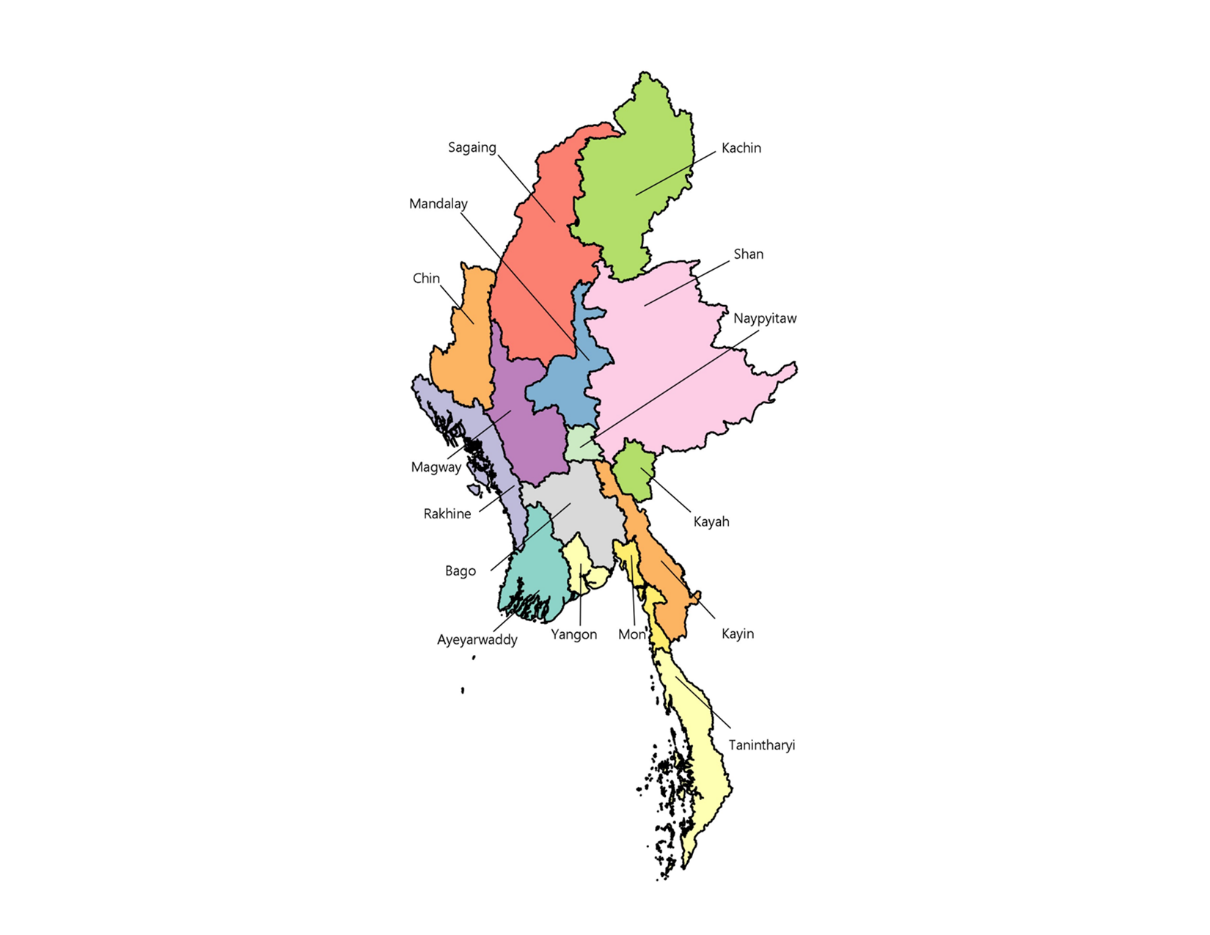


**S1 Fig. States and Regions of Myanmar**

Note: There are seven regions; they are areas that are predominately Burman (Bamar). There are seven states, they are home to Myanmar’s most politically and numerically dominant ethnic minorities. There is one union territory, Nay Pyi Taw, which we include as a state/region.

Source: Authors’ map based on data from MIMU Myanmar Information Management Unit. Access from: http://themimu.info/mimu-township-profiles-dashboard.


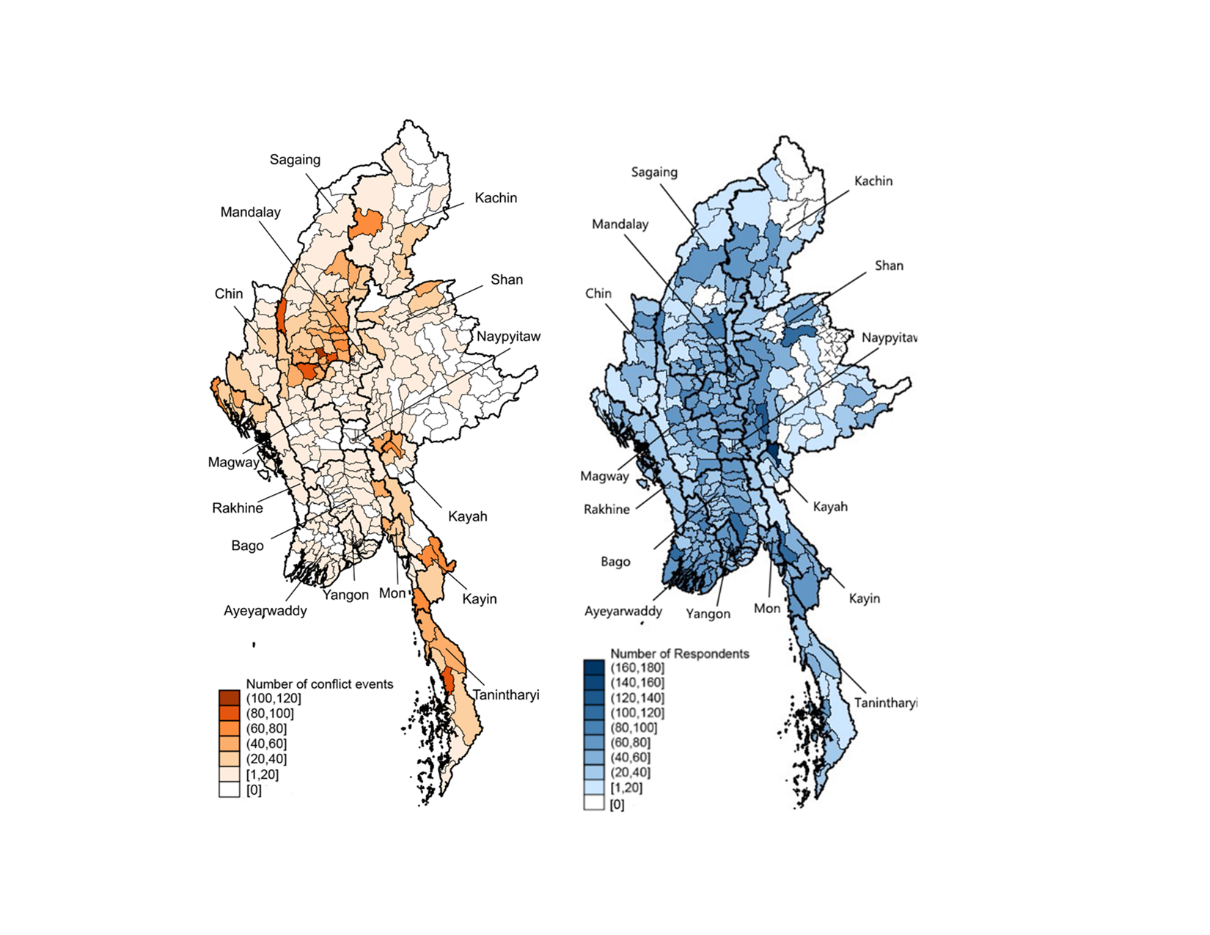


**S2 Fig. Interviews conducted in the fourth round of MHWS (left) and conflict events taking place during the months of data collection (right), by township**

Note: Stars indicate townships in Wa SAZ which were avoided for interviewing.

Source: Authors’ estimates from ACLED data (left) and authors’ map based on MHWS (right)


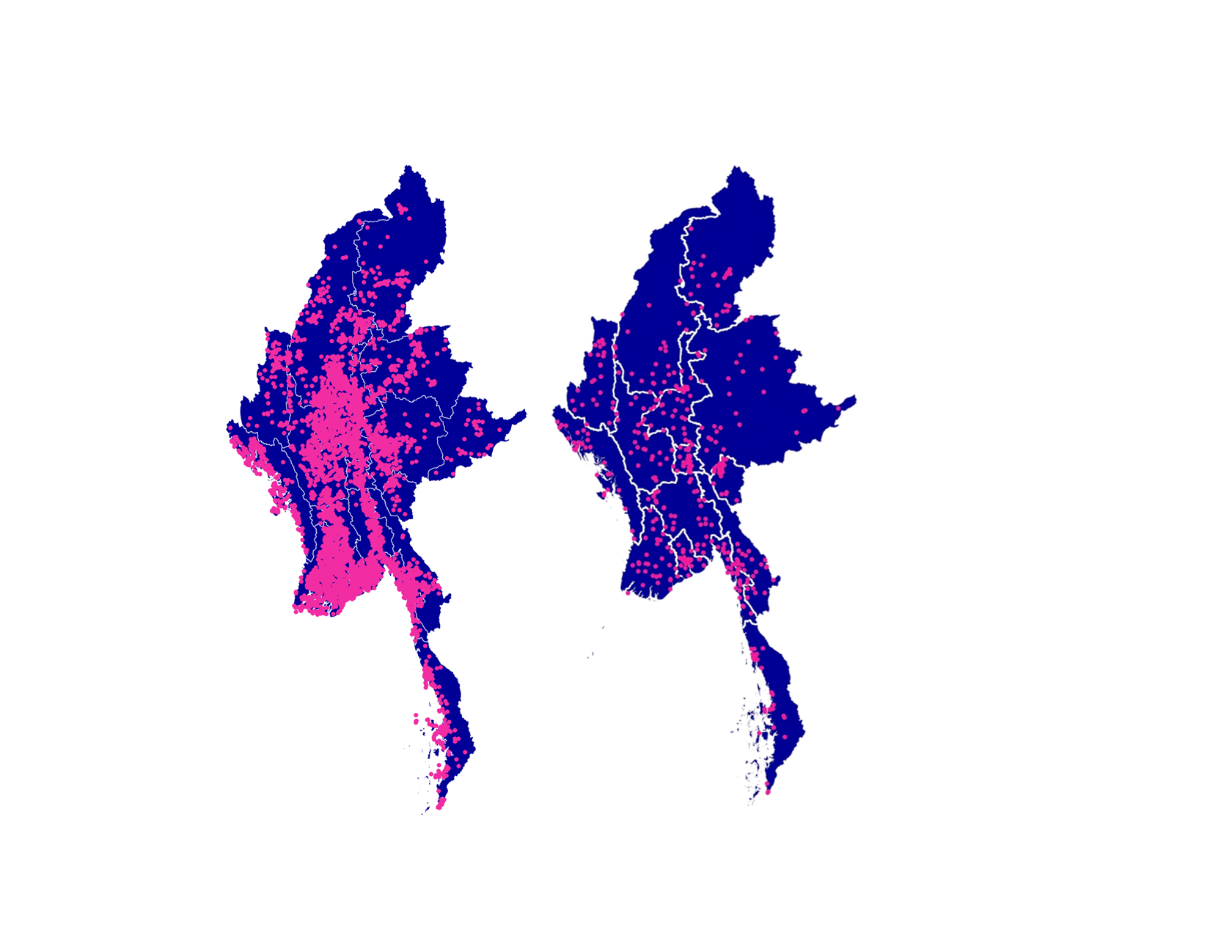


**S3 Fig. Comparison of townships of respondents of MHWS (left) and clusters where data were collected in the Myanmar DHS survey (right)**

Source: Authors’ map based on MHWS (left), and Myanmar DHS ppts for web [accessed online on February 16, 2022]

**
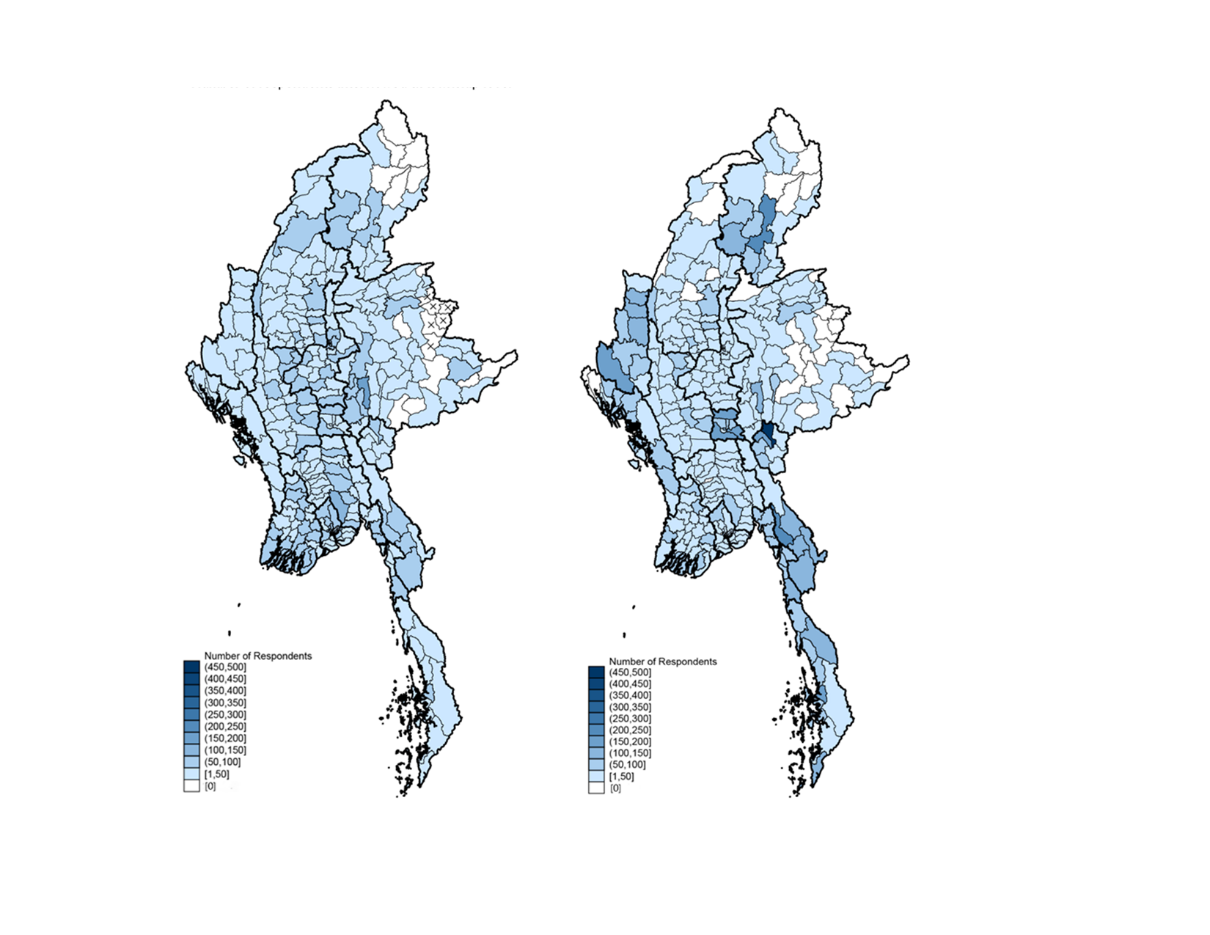
**

**S4 Fig. Comparison of townships of respondents of MHWS (left) and the Myanmar MLCS survey (right)**

Source: Authors’ map based on MHWS (left), and MLCS (right)
