# Supplementary material for: Can phone surveys be representative in low- and middle-income countries? An application to Myanmar
Source: PLoS One. 2023 Dec 22;18(12):e0296292. doi: 10.1371/journal.pone.0296292 (PMC10745169; doi:10.1371/journal.pone.0296292)
Supplement: Attachment — Submitted filename: PONE-D-23-25141.pdf [file pone.0296292.s003.pdf]

PLOS ONE

Phone surveillance – from scratch: National and subnational level data collection in Myanmar

--Manuscript Draft--

**Manuscript Number:**	PONE-D-23-25141
**Article Type:**	Research Article
**Full Title:**	Phone surveillance – from scratch: National and subnational level data collection in Myanmar
**Short Title:**	Phone surveillance – from scratch: National and subnational level data collection in Myanmar
**Corresponding Author:**	Joanna Van Asselt, Ph.D.<br>International Food Policy Research Institute<br>Washington, District of Columbia UNITED STATES
**Keywords:**	Phone Surveys, Fragile states, Conflict, High-frequency monitoring, Survey methods
**Abstract:**	For decades, in-person data collection has been the standard modality for nationally and sub-nationally representative socio-economic survey data. As the COVID-19 pandemic rendered in-person surveys impossible and unethical, the urgent need for rapid monitoring necessitated researchers and statistical agencies to turn to phone surveys. However, apart from pandemic-related factors, a variety of other reasons can render large segments of a population inaccessible for in-person surveys, including political instability, climatic shocks, and remoteness. Such circumstances currently prevail in Myanmar, a country facing civil conflict and political instability since the February 2021 military takeover. Moreover, Myanmar routinely experiences extreme weather events and is characterized by numerous inaccessible and remote regions due to its mountainous geography. We describe a novel approach to sample design and statistical weighting that has been successfully applied in Myanmar to obtain nationally and sub-nationally representative phone survey data. Our method results in a better geographical distribution compared to recent in-person survey efforts, including reaching respondents in areas of active conflict. Moreover, we minimize biases towards certain household and respondent characteristics that are usually present in phone surveys, for example towards well-educated or wealthy households, or towards men or household heads as respondents. Finally, due to the rapidly changing political and economic situation in Myanmar in 2022, the need for frequent and swift monitoring was critical. We carried out our phone survey over four quarters in 2022, interviewing more than 12,000 respondents in less than three months each survey. A survey of this scale and pace could only be possible on the phone. Our study proves the feasibility of collecting nationally and sub nationally representative phone survey data – from scratch, which is critical for rapid monitoring in any volatile economy.
**Order of Authors:**	Isabel Lambrecht
	Joanna Van Asselt, Ph.D.
	Derek Headey
	Bart Minten
	Patrick Meza
	Moe Sabai
	Thet Su Sun
	Hnin Ei Win
**Additional Information:**	
**Question**	**Response**
**Financial Disclosure**	This study was made possible by the support of the American people through the

Powered by Editorial Manager® and ProduXion Manager® from Aries Systems Corporation

Enter a financial disclosure statement that describes the sources of funding for the work included in this submission. Review the submission guidelines for detailed requirements. View published research articles from *PLOS ONE* for specific examples.<br><br>This statement is required for submission and **will appear in the published article** if the submission is accepted. Please make sure it is accurate.<br><br>**Unfunded studies**<br>Enter: *The author(s) received no specific funding for this work.*<br><br>**Funded studies**<br>Enter a statement with the following details:<br>• Initials of the authors who received each award<br>• Grant numbers awarded to each author<br>• The full name of each funder<br>• URL of each funder website<br>• Did the sponsors or funders play any role in the study design, data collection and analysis, decision to publish, or preparation of the manuscript?<br>• **NO** - Include this sentence at the end of your statement: *The funders had no role in study design, data collection and analysis, decision to publish, or preparation of the manuscript.*<br>• **YES** - Specify the role(s) played.<br><br>* typeset	United States Agency of International Development (USAID), under the terms of Award No. AID-482-IO-21-000x. Additional funding support for this study was provided by the CGIAR Research Program on Policies, Institutions, and Markets (PIM) and the Livelihoods and Food Security Fund (LIFT).
**Competing Interests**<br><br>Use the instructions below to enter a competing interest statement for this submission. On behalf of all authors, disclose any competing interests that could be perceived to bias this work—acknowledging all financial support and any other relevant financial or non-financial competing interests.<br><br>This statement is required for submission and **will appear in the published article** if	The authors do not have any competing interest regarding the publication of the manuscript. Publication of the manuscript will not affect funding, employment or other financial and non-financial interests of the authors, neither from IFPRI-affiliated nor from Myanmar Survey Research – affiliated authors.

the submission is accepted. Please make sure it is accurate and that any funding sources listed in your Funding Information later in the submission form are also declared in your Financial Disclosure statement.<br><br>View published research articles from *PLOS ONE* for specific examples.<br><br>**NO authors have competing interests**<br><br>Enter: *The authors have declared that no competing interests exist.*<br><br>**Authors with competing interests**<br><br>Enter competing interest details beginning with this statement:<br><br>*I have read the journal's policy and the authors of this manuscript have the following competing interests: [insert competing interests here]*<br><br>* typeset	
**Ethics Statement**<br><br>Enter an ethics statement for this submission. This statement is required if the study involved:<br><br>• Human participants<br>• Human specimens or tissue<br>• Vertebrate animals or cephalopods<br>• Vertebrate embryos or tissues<br>• Field research<br><br>Write "N/A" if the submission does not require an ethics statement.<br><br>General guidance is provided below. Consult the submission guidelines for detailed instructions. **Make sure that all information entered here is included in the Methods section of the manuscript.**	The IRB approved our submission to conduct the research activity named, Myanmar national phone survey. The date of our IRB approval was 09/20/2021. Our IRB application approval number is DSGD-21-0932.

Powered by Editorial Manager® and ProduXion Manager® from Aries Systems Corporation

**Format for specific study types**<br><br>**Human Subject Research (involving human participants and/or tissue)**<br>• Give the name of the institutional review board or ethics committee that approved the study<br>• Include the approval number and/or a statement indicating approval of this research<br>• Indicate the form of consent obtained (written/oral) or the reason that consent was not obtained (e.g. the data were analyzed anonymously)<br><br>**Animal Research (involving vertebrate animals, embryos or tissues)**<br>• Provide the name of the Institutional Animal Care and Use Committee (IACUC) or other relevant ethics board that reviewed the study protocol, and indicate whether they approved this research or granted a formal waiver of ethical approval<br>• Include an approval number if one was obtained<br>• If the study involved *non-human primates*, add *additional details* about animal welfare and steps taken to ameliorate suffering<br>• If anesthesia, euthanasia, or any kind of animal sacrifice is part of the study, include briefly which substances and/or methods were applied<br><br>**Field Research**<br><br>Include the following details if this study involves the collection of plant, animal, or other materials from a natural setting:<br>• Field permit number<br>• Name of the institution or relevant body that granted permission	
**Data Availability**<br><br>Authors are required to make all data underlying the findings described fully available, without restriction, and from the time of publication. PLOS allows rare exceptions to address legal and ethical concerns. See the PLOS Data Policy and FAQ for detailed information.	Yes - all data are fully available without restriction

A Data Availability Statement describing where the data can be found is required at submission. Your answers to this question constitute the Data Availability Statement and **will be published in the article**, if accepted.<br><br>**Important:** Stating 'data available on request from the author' is not sufficient. If your data are only available upon request, select 'No' for the first question and explain your exceptional situation in the text box.<br><br>Do the authors confirm that all data underlying the findings described in their manuscript are fully available without restriction?	
**Describe where the data may be found in full sentences. If you are copying our sample text, replace any instances of XXX with the appropriate details.**<br><br>• If the data are **held or will be held in a public repository**, include URLs, accession numbers or DOIs. If this information will only be available after acceptance, indicate this by ticking the box below. For example: *All XXX files are available from the XXX database (accession number(s) XXX, XXX.)*.<br>• If the data are all contained **within the manuscript and/or Supporting Information files**, enter the following: *All relevant data are within the manuscript and its Supporting Information files.*<br>• If neither of these applies but you are able to provide **details of access elsewhere**, with or without limitations, please do so. For example:<br><br>*Data cannot be shared publicly because of [XXX]. Data are available from the XXX Institutional Data Access / Ethics Committee (contact via XXX) for researchers who meet the criteria for access to confidential data.*<br><br>*The data underlying the results presented in the study are available from (include the name of the third party*	All the rounds of the Myanmar Household Welfare data are available on our website https://myanmar.ifpri.info/2022/09/06/myanmar-household-welfare-survey-mhws-round-1/. They can also be found on Harvard Dataverse at https://doi.org/10.7910/DVN/1R3F3U for round 1, https://doi.org/10.7910/DVN/LPNMTK round 2, https://doi.org/10.7910/DVN/GVJKAI round 3, and https://doi.org/10.7910/DVN/IKGJWF, round 4.

and contact information or URL).<br>• This text is appropriate if the data are owned by a third party and authors do not have permission to share the data.<br><br>* typeset	
Additional data availability information:	

Powered by Editorial Manager® and ProduXion Manager® from Aries Systems Corporation

Manuscript

Click here to access/download;Manuscript;manuscript_08_07_23.docx

Phone surveillance – from scratch: National and subnational level data collection in Myanmar

Isabel Lambrecht[1], Joanna van Asselt[1*], Derek Headey[1], Bart Minten[1], Patrick Meza[2], Moe Sabai[1], Thet Su Sun[2], Hnin Ei Win[1]

[1] International Food Policy Research Institute, Development Strategy and Governance Division, Yangon, Myanmar.

[2] Myanmar Survey Research, Yangon, Myanmar.

*Corresponding Author

E-mail: j.vanasselt@cgiar.org

Abstract

For decades, in-person data collection has been the standard modality for nationally and sub-nationally representative socio-economic survey data. As the COVID-19 pandemic rendered in-person surveys impossible and unethical, the urgent need for rapid monitoring necessitated researchers and statistical agencies to turn to phone surveys. However, apart from pandemic-related factors, a variety of other reasons can render large segments of a population inaccessible for in-person surveys, including political instability, climatic shocks, and remoteness. Such circumstances currently prevail in Myanmar, a country facing civil conflict and political instability since the February 2021 military takeover. Moreover, Myanmar routinely experiences extreme weather events and is characterized by numerous inaccessible and remote regions due to its mountainous geography. We describe a novel approach to sample design and statistical weighting that has been successfully applied in Myanmar to obtain nationally and sub-nationally representative phone survey data. Our method results in a better geographical distribution compared to recent in-person survey efforts, including reaching respondents in areas of active conflict. Moreover, we minimize biases towards certain household and respondent characteristics that are usually present in phone surveys, for example towards well-educated or wealthy households, or towards men or household heads as respondents. Finally, due to the rapidly changing political and economic situation in Myanmar in 2022, the need for frequent and swift monitoring was critical. We carried out our phone survey over four quarters in 2022, interviewing more than 12,000 respondents in less than three months each survey. A survey of this scale and pace could only be possible on the phone. Our study proves the feasibility of collecting nationally and sub nationally representative phone survey data – from scratch, which is critical for rapid monitoring in any volatile economy.

1. Introduction

Despite their cost-effectiveness and ability to access respondents in hard-to-reach places, the uptake of national phone surveys was limited prior to COVID-19 (Dillon 2012, Demombynes et al. 2013, Dabalen et al. 2016). With the onset of COVID-19 the implementation of phone surveys grew tremendously, mostly through necessity rather than desire. Three years into the pandemic, phone

surveys are no longer a rarity nor a novelty. Yet, they still seem to be considered "second-best" to in-person survey data collection. Whereas sampling and weighting procedures for generating nationally and sub nationally representative datasets are well established for in-person survey data collection, there is no such gold – or even silver – standard (yet) for phone survey data collection and phone surveys often get scrutinized for representativeness.

There are genuine concerns that those answering phone surveys might not be representative of all households or individuals in the population. First, not everyone owns a phone. Phone ownership is strongly associated with a range of factors, including geographical as well as household- and individual-level characteristics. Urban and suburban residents, and those living in well-connected areas with working infrastructure, including cell towers and electricity, are more likely to own phones (L'Engle et al. 2018; Lau et al. 2019). Moreover, mobile phone owners are typically better off, more educated, younger, and more male than non-owners (L'Engle et al. 2018; Lau et al. 2019; Gourlay et al. 2021). Second, non-response and refusal rates are much higher in phone surveys compared to in-person surveys and may also be linked to certain characteristics. Poorer people, farmers, or women may be less likely to answer a phone and could therefore be under-represented when randomly calling phone numbers (Gourlay et al. 2021).

Another challenge when setting up a phone survey is getting access to a comprehensive list of phone numbers. In the absence of any other feasible or affordable alternatives and considering the urgency of data collection needs during the early stages of the COVID-19 pandemic, many phone surveys relied on contact information from existing non-representative program-based surveys. Those that did organize national-level phone surveys mainly relied on random-digit dialing (RDD) (L'Engle et al. 2018), accessed contact information from households who were part of pre-COVID in-person survey datasets (Brubaker, Kilic and Wollburg 2021), or accessed lists of phone numbers from telecom providers in the country (Himelein and McPherson 2021).

Random digit dialing or calling respondents from telecom provider lists likely necessitates large amounts of phone calls to find respondents from these specific subpopulations - especially if regions are diverse in population size, phone ownership or response rates - thereby raising the cost of the survey quite substantially. Using contact information from in-person survey datasets may result in underrepresentation of certain geographies or segments of the population who have low mobile

phone ownership. Further, the use of sampling weights cannot always correct these biases to ensure representativeness – especially at the individual level (Brubaker et al. 2021, L'Engle et al. 2018; Lau et al. 2019).

However, in countries that are highly diverse in multiple dimensions (ethnicity, economic status, geography, and livelihoods), sub-nationally representative surveys are critically important for monitoring and evaluation, for program design and targeting, and for generating a more rigorous body of evidence for a wide range of policies and programs.

The objective of this paper is twofold. First, we highlight the potential of phone survey data collection as a modality to obtain nationally and sub nationally representative socio-economic data where face-to-face interviews are not feasible at a large scale. Phone surveys were pictured more prominently as a modality of data collection during a pandemic, mostly the recent COVID-19 pandemic but also for example during former Ebola outbreaks (Etang et al. 2020; Maffioli 2020). We demonstrate how phone surveys can be a powerful tool beyond pandemics, especially in fragile states. While there are obvious challenges with achieving representativeness of phone surveys, there are also under-appreciated advantages of phone surveys in countries beset by remoteness, conflict, and pandemic conditions (Maffioli 2020).

Second, the paper provides details on an innovative sample design and weighting strategy to minimize bias in characteristics typically associated with phone surveys, even when starting 'from scratch', i.e. when no representative phone number dataset is available. Our findings demonstrate that the pre- and post-survey methods we employed to address biases in phone surveys could serve as a valuable template for implementing sub-nationally representative surveys "from scratch" in other countries facing intricate governance and logistical challenges.

The example we bring is from a data collection effort in Myanmar. By early 2022, Myanmar was grappling not only with the economic and health consequences of two years of the COVID-19 pandemic, including its fourth wave (Omicron), but also facing severe political instability, armed conflict, and insecurity following the military takeover from the quasi-civilian government in February 2021. In such a difficult environment, accurate and frequent monitoring of the population's welfare, and with a sufficient granularity, is crucial for targeting scarce resources for

maximum impact and benefit to vulnerable populations. Yet, in-person nationwide survey data collection in Myanmar was, and is, infeasible.

We therefore organized a large-scale high frequency phone survey, representative at the national level, the urban/rural level, and the state/region level, called the Myanmar Household Welfare Survey (MHWS). Whereas previous socioeconomic surveys in Myanmar failed to reach significant parts of the country due to conflict (and were therefore perhaps not truly representative), the phone-based MHWS managed to survey the majority of Myanmar's townships, many of which have not been surveyed in recent times (e.g. northern Rakhine), and many of which were experiencing acute conflict and significant COVID-19 restrictions.

The paper is organized as follows. In section 2 we discuss the data and methods, including the sampling and weighting strategy for MHWS. Herein, we describe considerations made in the sample design, challenges in implementing data collection in relation to the sample, and calculations of the weights to reduce bias resulting from the composition of the final sample. In section 3 we report sample characteristics of the survey and compare MHWS sample characteristics to other recent nationally representative datasets. In section 4 we discuss attrition in our sample over the four rounds of data collection take took place in 2022. We conclude in section 5.

2. Data and methods

The first round (round 1) of MHWS data was collected from 12,100 households between December 17th, 2021, and February 13th, 2022. This was followed by a second round (round 2) in April-June 2022, a third round (round 3) in July-August 2022 and a fourth round (round 4) in October-December 2022. The objective of the survey was to collect data on a wide range of household and individual welfare indicators–including wealth, poverty, unemployment, food security, diet quality, subjective wellbeing, and coping strategies. The aim of the MHWS was to represent the population living in conventional households, similar to the usual target population of nationally representative datasets that collect data through face-to-face interviews. MHWS respondents could be any household member aged 18-74 years old. The lower limit of 18 years old was purposively chosen as childhood legally ends at 18 years old in Myanmar. The upper limit of

74 years old was chosen with the intention of minimizing communication and recall errors that we expected to be common among respondents of high age. Below we distinguish three parts of the sampling process: (i) the original pre-survey sample design, (ii) the MHWS sample, and (iii) the calculation of sampling weights.

2.1 The MHWS sample design

The MHWS intended to interview 12,790 respondents. The sample was distributed proportional to the population size in each State and Region based on the population data of the 2014 Census (DoP, 2015), but with a minimum 240 respondents for the two states with the lowest population size (i.e., Kayah and Chin State) (S1 Table). The MHWS sample design was similar to the 2015-2016 Myanmar Demographic and Health Survey (MDHS; 12,500 households) and the 2017 Myanmar Living Conditions Survey (MLCS; 13,730 households), both of which aimed to be representative of each state/region and of rural and urban areas of Myanmar as a whole.

The implementation of the MHWS occurred in collaboration with Myanmar Survey Research (MSR), a private survey research company based in Myanmar. MSR owns a database of 280,274 phone numbers of adults who consented to be contacted for future participation in phone survey data collection, including geographical information of the township of residence of the respondent. The first step in selecting phone numbers for interview was the development of a master phone number database. This master database was constructed as a "long list" for final survey sample selection and contained four times the actual number of target interviews (to account for non-response).

To create the master database all phone numbers were stratified at the township level, which is a smaller geographical unit within each state or region, numbering 330 in total. The final amount of phone numbers in the master database were proportional to the population size in each township, based on the information in the 2014 Census data. The township proportionality structure of this master database was designed to minimize the risk of oversampling respondents who live predominantly in well-connected and wealthier townships. Without a deliberate attempt to achieve such a spatial spread, a random selection of phone numbers risks reaching respondents who are clustered in urban areas, in localities with better infrastructure and with higher levels of asset

ownership and located in more connected geographical areas within a state. For Myanmar, not achieving a broad spatial spread could also mean not reaching townships that are under control of ethnic armed organizations (which are often either less connected or using phone numbers of neighboring countries). While we did not insist on having an exact proportional balance of interviews at township level in the final sample, the survey company did strive to achieve such balance to the extent possible.

Another concern we tried to mitigate was the likely underrepresentation of women, rural residents, people with lower levels of education and farmers. Such underrepresentation is often reported in phone survey samples (Brubaker et al. 2021; Gourlay et al. 2021). We therefore set the following minimum targets at State/Region level (S2 Table):

1. Gender (female): Half of all respondents should be female.
2. Location (rural): Respondents with rural residence, proportional to the population in conventional households based on the 2014 Myanmar Census Report.
3. Education (lower-educated): Respondents who completed at most primary school level. The target was calculated based on the percentage of adults in conventional households aged 25 years and over by highest level of education completed based on the 2014 census data. This percentage was then adjusted downward first to correct for the age range of our respondents (18 to 74 years old) and thereafter to account for shifting age cohorts between the time of census data collection (2014) and the start of our survey (2021).
4. Household livelihood (farming): Respondents living in a household where crops were harvested in the past 12 months. The share of farmer households was calculated based on the same question in the nationally representative 2017 MLCS, but an additional 5 percent buffer was added. This oversampling of households with farm livelihoods was primarily because they are a key group of interest, and we planned follow-up surveys specifically for farm households.

In practice, the approach adopted to achieve these targets was as follows. After explaining the purpose of the study and obtaining informed consent, the respondent first answered survey screening questions related to the quota (age, gender, location, education level and household livelihood). Based on this information, it was assessed whether the interview quota for respondents

with these characteristics were already met, and if so, the respondent was explained that s/he would not be interviewed at this time but may be contacted again in the future.

There was no instruction to the interviewers that the owner of the phone number him-or herself should respond to the interview questions. In some cases, another person answered the call and agreed to be interviewed, while in other cases, the person who answered the call handed over the phone to another household member to be interviewed. Additionally, enumerators were clearly instructed that any household member between 18 and 74 years old was eligible to be interviewed (i.e., they did not need to target the household head for the interview). If the respondent's age was too low or high to be interviewed, s/he was asked to hand over to another household member.

Sample deviations were expected given that a large share of the population was directly or indirectly affected by conflict, disruptions to telecommunication services, frequent power outages, economic distress, and displacement during the periods of data collection. While the final sample in round 1 did not fully achieve the attempted sample targets and sizes, in round 4 the sample targets were met (S3 Table)[1]. In states and regions where targets could not be achieved after reaching out to all phone numbers in the master dataset, the survey company attempted to reach respondents from the respective townships in their panel database who were not selected in the master dataset. Even so, attempted targets could not always be met. The most severe problems of falling short of pre-determined targets in round 1 were related to two issues. First, target gaps occurred in the two smallest states (Chin and Kayah). Reaching targets there was more challenging than in other places due to a combination of higher targets relative to the population size (i.e. the intended oversampling), the remoteness of these areas (particularly in Chin State), and the fact that these areas were highly affected by active conflict. Second, it proved difficult to reach the quota of respondents with low levels of education in several States and Regions. Despite these shortcomings in round 1, in round 4 we slightly altered our data collection method, so that every week throughout the three months of data collection, households were called in each state/region. This consistency allowed us to exceed our sample targets for Kayah and Chin, as well as meet our low-education target.

2.2 The construction of MHWS sampling weights

Two main steps were used to construct sampling weights: (a) we calculated a set of basis weights that makes the required adjustments related to the sample target characteristics; (b) we applied a maximum entropy approach to further minimize residual bias in observed characteristics in terms of wealth and household composition. We re-calculate these weights in each round, following the strategy laid out below, to ensure representativeness of the data in each round.

2.21 Basis household weights

For sample estimates to be representative of the population in terms of the sample characteristics we developed the basis of the household-level weights using three main steps:

1. Apply an expansion factor: We weight households for their probability of occurring in the sample, based on the newly released 2019 ICS information of the number of households in each urban or rural location of each state and region. This step ensures representativeness at state/region level and the share of households in rural (urban) locations in each of these States and Regions.
2. Adjust for oversampling of farm households: In rural areas of each state and region we proportionally adjust the household weight of farm and non-farm households to have the same percentage of farm households as found based on MLCS estimates. No further correction for livelihoods was made at the urban level given the low number of farmers in that category.
3. Weight for education level of the respondent: We proportionally re-weight households based on the level of education of their respondent (i.e., to adjust for oversampling of more educated respondents).

Step (3) is complicated by the strong association of household and respondent characteristics with educational attainment. Weighting factors for step (3) were therefore calculated based on the share of adults with low education aged 13-69 years old in 2017 (i.e., who would be 18-74 years old in 2022), by relation to the household head (head and spouse, versus other household members), by urban/rural location, and household livelihood within each State or Region. Analyses

of MLCS show no significant difference between the share of men and women who have low educational attainment, so weighting based on gender of the respondent did not seem warranted.

2.22 Entropy-adjusted household weights

Further bias in our sample may appear in terms of characteristics that are independent of the characteristics already stratified and weighted for. To correct this kind of residual bias, we rely on the maximum entropy approach (Wittenberg, 2010; Hainmueller, 2012). This approach can be used to generate or adjust weights to match averages and totals of pre-selected indicators. It is increasingly being used to calibrate survey data to various population totals and was also used to calibrate survey weights of several of the World Bank's high frequency phone surveys initiated during the COVID-19 pandemic (The World Bank, 2021).

Calibration of the survey weights should only be based on characteristics that are time-invariant or slowly changing over time. The most recent nationally representative dataset (partially) available to these authors was the 2017 MLCS data, collected five years prior to the current MHWS. In a country that was transforming extremely rapidly prior to 2020 (including for example a massive surge in mobile phone ownership), and then was set back by a series of extreme social and economic shocks (i.e. a pandemic and conflict), many characteristics have likely changed. Moreover, a phone survey is typically short in nature and thus also limited in terms of available indicators to match between datasets. Moreover, while the 2018 Intercensal Survey (ICS) data was collected right before the onset of the pandemic, we can only access its reports but not the raw data itself.

The maximum entropy procedure is applied using the basis weights calculated in step (a) and included constraints to maintain the total number of households in each State or Region and by Urban and Rural location (based on the 2020 estimate). Two additional sets of constraints were added related to wealth: (i) agricultural land owned (in five categories), based on the distribution of the 2017 MLCS data; and (ii) housing type (apartment, bungalow/house, semi-pucca house, or other) among urban households, based on the reported 2020 ICS information. Finally, we set constraints for household composition. More specifically, this approach adjusts for households where all adults are women (women-adult-only households, WAH) in rural and urban areas separately, based on the 2017 MLCS data.

Finally, we also develop population weights and individual weights. The population weights are calculated as the household weights multiplied by the number of household members reported by each respondent. Individual weights are developed for representation of individual-level data among the adult population (aged 18-74 years old). Individual weights are therefore also straight-forwardly calculated as household weights multiplied by the number of adults in the household.

2.4 Ethics

All interviews were conducted by phone with respondents who consented to be interviewed during earlier recruitment by the survey company. Each phone call started with a short introduction of the study and informed consent. In addition, a screening question inquiring about the age of the respondent was asked to ensure no minors were interviewed. An institutional ethics approval was obtained from the International Food Policy Research Institute's institutional review committee prior to piloting and implementing the survey.

2.5 Demographic and socioeconomic comparability to previous surveys

We assess the performance of the dataset and sample weights in reflecting the spatial and socio-economic diversity of the country through comparisons with findings from three sources of survey data; (1) the 2015-2016 Myanmar Demographic and Health Survey (MDHS); (2) the 2017 Myanmar Living Conditions Survey (MLCS); and (3) the 2019 Inter-Censal Survey (ICS). Even though MDHS data are less recent than the other datasets, the MDHS is known as the national socioeconomic survey most successfully reaching parts of the country that are difficult to access. Hence, it is interesting to compare MHWS with MDHS in terms of spatial achievement. The publicly available MDHS dataset does not include variables allowing us to identify the township of the respondent and we therefore rely solely on the information available in their reports.

The MLCS data was the last nationally and subnationally representative socioeconomic survey conducted in-person in Myanmar (CSO, UNDP & World Bank 2019a, 2019b). This is the only dataset that allows us to identify the township where each household resides. The 2019 Inter-Censal Survey (ICS) is the most recent and relevant representative nationwide survey effort

(December 2019 – February 2020), yet we do not have access to the dataset itself and thus are restricted to the information available in the ICS report (DOP, UNFPA 2020). Information on conflict is taken from ACLED (https://acleddata.com) and includes the number of battles, explosions, and violent events.

We compare unweighted MHWS sample means, weighted MHWS estimates and other nationally-representative (ICS or MLCS) household-level and individual-level estimates to explore biases in wealth and socio-economic status, and residual differences with other nationwide estimates after weighting. ICS data was collected during the same time of year as the MHWS, which is relevant for comparing indicators affected by seasonality, such as the sources of drinking water. We similarly compare individual-level estimates of adults' ages, roles in the households and education with the MLCS dataset. Note that options to explore concerns related to biases in the MHWS is somewhat limited given the paucity of indicators that are expected to be similar and that are available in both datasets.

3. Results

3.1 Data collection in conflict-affected areas

The geographical coverage of the MHWS is better in comparison to former national surveys. In round 1 respondents were reached in 310 of the 330 townships in Myanmar *(de facto* only 324, as we were legally restricted to excluding six townships in Wa SAZs). Fig 1 shows a map of respondents interviewed in round 1, whereas appendix S1 Fig contains a map of enumeration for round 4. By round 4, the number of townships represented declined to 303. Between R1 and R4 conflict intensified across the country. At the same time, blackouts increased in frequency and duration. The list of the 20 non-surveyed townships in round 1 consists of excluded townships (six townships in Wa SAZs), townships with very low population sizes or townships that are highly inaccessible–even by phone (Fig 1 and S4 Table). In total the population of these townships consists of about 1.6 percent of the total 2019 population in conventional households of Myanmar, but about half of this non-enumerated population is from the excluded townships in Wa SAZ (Shan State). Hence from the perspective of the target survey population (i.e. excluding Wa SAZ), in R1

we only fail to reach townships that contain as little as 0.76 percent of the target survey population (S4 Table).

Fig 1. Interviews conducted in the first round of MHWS (left) and conflict events taking place during the months of data collection (right), by township

Note: stars indicate townships in Wa SAZ which were avoided for interviewing.

Source: Authors' estimates from ACLED data (left) and authors' map based on MHWS (right)

While in both round 1 and round 2 we surveyed 310 townships, in round 3, three additional townships were not enumerated, all of which have very low population sizes. In round 4, three additional townships were not enumerated, this time due to intense fighting in Sagaing and Kayah. Therefore, despite ongoing intense conflict, after four rounds, we only failed to reach townships that contain 1.46 percent of the target survey population (S4 Table).

The MHWS geographical spread of 310 townships is better than face-to-face national-level survey efforts of similar sample sizes, such as the 2015-16 MDHS that reached 250 out of the 413 townships classified at that time (DoP, 2020) and the 2017 MLCS that reached 296 townships of 330 townships. See S2-S3 Figs and S1 Table for details. This greater township coverage is in part due to the two-stage survey design setup of face-to-face surveys, which cluster typically between 12 and 30 survey households within each enumeration area to reduce on transport and other logistical costs. These cost savings are necessary for face-to-face surveys but not for phone survey interviews. An additional drawback is that clustering strategies used in in-person surveys reduces precision of indicator estimates.

All existing nationwide face-to-face survey efforts in Myanmar have been significantly hampered by inaccessibility, insecurity, and travel restrictions. An estimated 1.2 million people (about 2.3 percent of the total population) could not be enumerated during the 2014 Census (DoP, 2015). The 2019 ICS excluded eight townships in Shan State from the ICS sample frame due to expected inaccessibility (this includes six townships in Wa SAZ that we also excluded, one in Kokang SAZ that we also did not reach, and Laukkaing which we reached). Out of the remaining ICS enumeration areas, 8 percent were not enumerated due to operational difficulties. In the past decade especially, Rakhine State suffered from extreme insecurity resulting in survey enumeration efforts in the state being severely hampered. It is estimated that during the 2014 Census about

31 percent of the population of Rakhine State could not be enumerated (DoP, 2015). In 2017 the MLCS team was unable to collect data in two townships of Rakhine State, while the 2019-2020 ICS was unable to reach about 74 percent of the selected enumeration areas in Rakhine State (DOP, UNFPA 2020). These figures clearly demonstrate the challenges of implementing face-to-face surveys in Myanmar, even at a time when conflict was less widespread compared to in 2022.

Fig 1 compares a map indicating violent events taking place during the period of MHWS R1 data collection (based on ACLED data) with MHWS respondents reached in each township. S1 Fig in the supporting information contains the maps for round 4 of MHWS. This comparison shows how we still managed to reach a reasonable number of respondents even in most of the conflict-affected townships. MHWS successfully reached respondents in all 17 townships of Rakhine State. Fewer conflict events were experienced in Rakhine State in the recent year compared to the years prior, but nevertheless insecurity did not cease entirely.

We further compare the relation between conflict incidence and conflict intensity with enumeration intensity for MHWS and MLCS in Table 1 and S5 Table. Conflict intensity was measured through the monthly average of conflict events in non-enumerated townships during each survey's respective enumeration period and expressed relative to the population size (per 10,000 households). For each survey, we distinguish between underrepresented townships (≤ 7 respondents / 10,000 households), normally represented townships (7-14 respondents / 10,000 households), overrepresented townships (>14-22 respondents / 10,000 households) and largely overrepresented townships (>22 respondents/ 10,000 households). Note that a perfectly proportional distribution (obtained by dividing the total sample size by the number of households in the country) suggests 12 enumerated households per 10,000 households in MLCS and 11 enumerated households per 10,000 households in MHWS.

We find no patterns of exclusion or under-representation of townships who suffered from conflict in MHWS, but we do find this in the MLCS. Non-enumerated MHWS townships experienced similar or lower conflict incidence and intensity than enumerated townships (Table 1). During R1 of MHWS only one out of twenty non-enumerated townships experienced conflict. This is much lower than the national average, given that nationwide 239 of the 330 townships suffered from conflict in that period. In contrast, conflict incidence and intensity is much higher in MLCS non-enumerated

townships compared to MLCS enumerated townships. Eleven out of 35 non-enumerated townships experienced conflict, whereas only 27 of the remaining 297 enumerated townships did so.

Table 1. Monthly average of violent events and experience of any violence during survey period, at township level and relative to population size

Township groups based on number of households interviewed per 10,000 households	MHWS			MLCS		
	# conflicts per 10,000 households	% townships with conflict	*# townships*	# conflict per 10,000 households	% townships with conflict	*# townships*
No respondents (0)	3.08	18%	23	2.38	31%	*35*
Underrepresented (≤7)	6.06	62%	39	0.00	1%	*69*
Normal representation (7-14)	4.09	72%	212	0.07	7%	*134*
Overrepresented (14-22)	7.52	79%	43	0.28	19%	*31*
Largely overrepresented (>22)	16.16	81%	12	0.25	18%	*60*

Note: One area was reclassified from one to two townships recently and we keep them together for this comparison table.
Source: the authors' estimates from MHWS, MLCS and ACLED (number of battles, explosions, and violent events)

Table 1 and S3 Fig also demonstrate that MHWS reaches more townships and achieves a more even spread (i.e. less townships with no respondents and less townships with a disproportionally high amount of respondents). The twelve townships with higher-than-average representation in MHWS include four townships in Chin State and four townships in Kayah State. These States were intentionally oversampled given the small population size in these States, yet they are also two States highly affected by conflict. The MLCS similarly disproportionally interviews households from all townships in Chin and Kayah State, likely due to a combination of intentionally oversampling the smallest states, potentially aggravated by the clustered sample design.

3.2 Household composition and wealth: comparisons with other nationally representative data

The household composition in MHWS is similar to the composition observed in MLCS. Table 2 shows the comparison of these characteristics from the pooled sample, and the accompanying appendix S6 table demonstrates the differences between unweighted and weighted MHWS estimates. We find a modest difference with MHWS households having more adults aged 15-64 years old, and fewer children and senior adults. Even though half of the phone survey respondents were female, the phone survey sample consisted of slightly over half of the estimated share of women-adult-only households (WAH) in the MLCS dataset. However, this was successfully corrected for with our weighting strategy.

Table 2. Household composition comparisons between the MHWS and the MLCS

	National		Rural		Urban	
	MHWS	MLCS	MHWS	MLCS	MHWS	MLCS
child <5y	24%	29%	22%	25%	24%	30%
child 5-14y	48%	51%	44%	44%	50%	53%
adult 15-64y	100%	97%	100%	97%	99%	97%
senior >= 65y	22%	25%	21%	27%	23%	24%
# children <5y	0.27	0.34	0.25	0.29	0.28	0.35
# children 5-14y	0.70	0.80	0.62	0.67	0.72	0.85
# adults 15-64y	3.06	2.82	3.12	2.93	3.04	2.78
# seniors >= 65y	0.28	0.32	0.25	0.35	0.29	0.30
Women adults only [a]	9%	9%	10%	10%	9%	9%

[a] These are households with women adults (aged >14 years old) but without male adults (aged >14 years) in the household.
Notes: the estimates used the pooled sample from R1-R4, which has 49,294 observations.
Source: the authors' estimates from MLCS and MHWS.

Furthermore, the wealth indicators available from MHWS closely match those of MLCS and ICS, as shown in in Table 3. It is possible that some differences could be explained by subtle differences in phrasing and administration of survey questions, but this caveat aside, the set of indicators we present here are broadly comparable. The MHWS estimates approximate those for ICS in most housing characteristics – including those that we did not control for in the weighting strategy (i.e. tenure of dwelling, type of floor and improved source of drinking water). Compared to the ICS, estimates from the MHWS show a slightly lower prevalence of households in an owned or freely provided house, fewer households with an improved floor, and fewer households with improved sources of drinking water. This suggests that the MHWS survey, if anything, would be biased towards less rather than more wealthy households, although we could be picking up some of the genuine welfare losses incurred by the severe economic shocks of 2020,2021, and 2022.

Table 3. Wealth indicators comparing MHWS (pooled sample and weighted) and ICS and MLCS survey findings, in percentage of households

	National		Urban		Rural	
	MHWS	MLCS/ ICS	MHWS	MLCS/ ICS	MHWS	MLCS/ ICS
Agricultural land owned[a]						
0 acre	63	63	90	92	53	51
]0-2] acre	10	10	4	3	12	13
]2-4] acre	8	9	2	2	10	12
]4-7.5] acre	9	8	2	2	11	11
>7.5 acre	10	10	2	2	13	13
Type of dwelling[b]						
Wood/bamboo	67	67	48	47	74	75
Semi-pucca house	14	13	18	18	12	11

Bungalow	12	11	16	16	10	8
Apartment	5	6	17	17	1	1
Hut (2-3y)	2	3	1	1	2	4
Hut (1y)	1	1	0	0	1	1
House ownership						
Owned/free	90	93	74	81	96	98
Rented	9	7	25	19	3	2
Squatter	0	n/a	1	n/a	0	n/a
Camp, shelter	0	n/a	0	n/a	0	n/a
Improved floor	71	78	79	90	68	74
Improved drinking water source	78	82	93	92	72	78

[a] The comparison dataset for farm size is MLCS
[b] The comparison for housing indicators is the ICS.
Note: the estimates used the pooled sample from R1-R4, which has 49,294 observations.
Source: DoP and UNFPA (2020), and the authors' estimates from MLCS and MHWS.

A few points are also noteworthy from the weighted and unweighted comparisons reported in S6 Table. First, comparisons of unweighted and weighted MHWS indicators shows that MHWS likely interviewed too many households who own larger areas of agricultural land, potentially indicating a bias in our sample towards more wealthy farm households that was not corrected for by our sampling targets (e.g. education). Second, urban sample respondents less often reside in apartments (11 percent versus 17 percent in ICS) and more often reside in wood/bamboo houses (55 percent versus 47 percent in ICS). This indicates a potential bias in our urban sample towards fewer wealthy households. We corrected for these indicators in our weighting strategy, so this bias no longer appears in the weighted estimates.

3.3 Are respondents representative of the adult population?

Given that several indicators include individual-level - rather than household-level - characteristics, it is relevant to question the representativeness of the respondents for Myanmar's adult population. Table 4 shows comparisons of education level, age, relation to household head and gender between the adult-level weighted MHWS estimates, and the weighted 2017 MLCS adult population. Additionally, the comparison of unweighted and weighted MHWS estimates is shown in appendix S7 Table. We assume that using the information captured in the household roster in a well-conducted national phone survey, such as the MLCS, allows one to confidently estimate key characteristics of all individuals living in conventional households (provided also that

the correct weights are applied and ignoring for the moment the potential issues of non-representativeness of the MLCS).

Table 4. Adult population (aged 18-74 years) characteristics in the MHWS pooled sample and weighted and MLCS, in percentage

	National		Urban		Rural	
	MHWS	MLCS	MHWS	MLCS	MHWS	MLCS
low education level	58	58	36	38	67	67
junior: 18-24	18	17	22	17	17	17
middle: 25-49y	60	53	62	53	59	54
senior: 50-74y	22	30	16	30	24	29
head	34	34	30	32	35	34
Spouse of head	23	25	21	23	24	26
Child of head	33	34	37	36	31	34
Other relation	5	7	8	9	4	5
Female	53	54	53	55	53	54

Notes: Demographic variables from the MLCS dataset are based on the information from all household members aged 18-74 years included in the household roster. The estimates used the pooled sample from R1-R4, which has 49,294 observations.
Source: Authors' estimates from 2022 MHWS and 2017 MLCS

We find a reasonable approximation of basic respondent characteristics of MHWS compared to MLCS in terms of education level, age, the relation to the household head and gender (Table 4). Our dataset does not suffer the same shortcomings as noted by Gourlay et al. (2021) and Brubaker et al. (2021), where respondents are disproportionally male and household heads. The share of youth in our sample is a good approximation of the share of youth in the general population, contrary to findings from other phone survey studies who either find an overrepresentation (Henderson and Rosenbaum 2020) or underrepresentation of youth (Brubaker et al. 2021). However, we find a higher share of middle-aged people (25-49 years old) and a lower share of older people (age category 50-74 years old). This bias is particularly present among urban respondents and to a lesser extent among rural respondents.

S6 Table shows that differences between adult respondents in the MHWS and the adult population in the MLCS further reduce after weighting the MHWS data rather than aggravate as in Brubaker et al. (2021). Despite setting (but not fully achieving) sample targets for respondents with lower levels of education, we observe an overrepresentation of respondents with higher education levels in the sample. However, this issue is resolved after weighting given that our weights are calibrated for education.

4. Attrition

The MHWS survey was initially designed to be a panel survey, but inevitably suffered from attrition. Between round 1 and round 4 of the data collection, the spread and intensity of conflict, electricity blackouts, and damaged phone lines increased, and as a result, non-response was high among our previously interviewed respondents. For example, from round 1 to round 2, 36 percent of respondents left the sample. Attrition decreased between round 2 and round 3 as well as between round 3 and round 4, with 25 and 24 percent of respondents leaving the sample after those respective rounds. We show cross-tabulations of the households who remain in the sample between any two different rounds in Table 5.

To replace the households that dropped out of the survey, the survey team called new households. The households were selected randomly from the phone database, the same townships as the attrition households, and retained if they had similar characteristics to the attrition households in terms of urban/rural, gender, farm, and low education. If the survey team could not meet those criteria, they called households with similar characteristics from the same state/region. It is important to note that some of the households that were not interviewed in the next round, were interviewed in subsequent rounds, for example 13 percent of the round 1 respondents that were not interviewed in round 2, were interviewed in subse[illegible]t rounds.

Table 5. Crosstabulation of number of households interviewed in four survey rounds

	R1 (N=12,100)	R2 *(N=12,142)*	R3 *(N=12,128)*	R4 *(N=12,924)*
R1 (N=12,100)	3,725	7,799	6,567	5,172
R2 (N=12,142	7,799	1,280	9,054	7,189
R3 *(N=12,128)*	6,567	9,054	8,28	9,200
R4 *(N=12,924)*	5,172	7,189	9,200	3,724
All rounds (1-4)	4,831	4,831	4,831	4,831

Source: Authors' estimates from 2022 MHWS

In Table 6, we explore differences between households that dropped out of the sample for each round (attrition households) and households that remained in the sample (panel households). We also investigate the differences between attrition households and replacement households. We

present the marginal effects from probit regressions that explore the association of individual, household, and geographic characteristics on whether a household left the sample. In column (1) we analyze attrition from round 1 to round 2 by using round 1 characteristics and a dummy outcome variable for whether the household is still in the sample in round 2 (1 if attrition, 0 if still in the sample). In column (2) we analyze attrition from round 2 to round 3 by using round 2 characteristics and a dummy for if the household is still in the sample in round 3. In column (3) we analyze attrition from round 3 to round 4 by using round 3 characteristics and a dummy for if the household is still in the sample in round 4. In column 4 we compare households that left the sample in round 1, 2, or 3, with households that remained in the sample in every round.

While in some rounds, households with certain characteristics were more likely to drop out of the sample, in our analysis we focus on consistent predictors across the three rounds. Overall, households with a female survey respondent were less likely to leave the sample. Respondents with higher education were more likely to drop out of the sample. In terms of household characteristics, household size, number of dependents, or whether the household had only female members, were not significant predictors of attrition. Further, households who moved more recently were not more likely to drop out of the sample. While having a household of improved build was not associated with attrition by round, attrition households were less likely to have an improved house than households that remained in the sample in every round. Finally, households with improved electricity were less likely to attrite in most rounds and compared to panel households in all three rounds.

For the most part, there was no association between attrition households and their main source of income. The only exception was that compared to farm households, households whose main source of income was salaried work were less likely to drop out of the sample between round 1 and round 2 and compared to the households that remained in the sample in every round. While between round 1 and round 2, compared to households in the lowest income quintile, households in higher income quintiles were not more likely to leave the sample, in round 2 and round 3, wealthier households do appear more likely to attrite, though the relationship does not appear to be linear. On the other hand, compared to households that remained in the sample in every round, households in the three highest income quintiles were less likely to attrite. Importantly, shocks

appear to have no impact on attrition. In fact, households who reported feeling insecure were less likely to attrite in every round. Finally, remote households were more likely to attrite across the rounds. Overall, it appears that wealthier households were more likely to remain in the sample, but only by a very marginal amount.

We also investigate the differences between replacement households and attrition households in columns (5-6). In column (5) we analyze attrition from round 2 to round 3 by using round 2 characteristics. We define a replacement household as a household who joins the sample in round 2 and was not present in round 1. In column (6) we analyze attrition from round 3 to round 4 by using round 3 characteristics. We define a replacement household as a household who joins the sample in round 3 and was not present in round 2.

Compared to replacement respondents, between round 3 and round 4, attrition households were less likely to be low educated. Attrition households were significantly more likely to have moved in the past two years compared to replacement households. This suggests that we struggled to add displaced households to our survey, despite the increase in displacement in Myanmar over the year. There were not clear patterns between attrition and replacement households in terms of agricultural land holdings and source of income. Attrition households were more likely to be in the wealthiest income quintile, compared to the poorest. Finally, attrition households were less likely to experience a climatic shock, compared to replacement households. This suggests that other than struggling to capture displaced households, our sampling strategy successfully replaced attrition households with households with similar characteristics.

Table 6. Analysis of attrition and replacement in four survey rounds

	Panel households				Replacement households	
	R1	R2	R3	R1,R2,R3	R2	R3
Respondent's age (#)	-0.00***	-0.00***	-0.00***	-0.01***	0.00***	0
Respondent is female (0/1)	-0.01	-0.02**	-0.03***	-0.04***	-0.02	-0.01
Respondent has at most primary education (0/1)	0.03***	-0.01	0.02**	0.02**	0.02	-0.09***
Dependents (#)	0	0	-0.01	0	0.02**	0.01
Household size (#)	0.01**	0	0	0.01***	0.02***	0
Women only household (0/1)	0.01	-0.03	0	0.01	-0.02	0
Moved to current village <= 2 years ago (0/1)	0.01	0.03	0.02	0.01	0.14***	0.10***
Improved house [a] (0/1)	-0.02	-0.01	-0.01	-0.02***	-0.03	0.03
Household has electricity connection [b] (0/1)	-0.04***	-0.02*	0	-0.03***	0.01	0

Agriculture land > 0-2 acre vs no land	0.02	-0.04***	-0.01	0	-0.04	0.03
Agriculture land 2-4 acre vs no land	0.01	-0.03**	0.01	0	-0.03	0.06**
Agriculture land 4-7.5 acre vs no land	0.02	-0.05***	0.02	-0.01	-0.06**	0.09***
Agriculture land >7.5 acre vs no land	-0.01	-0.03**	0.01	-0.03**	0.01	0.11***
Farm wage vs own farm	0	0.01	0	0.01	0.05*	-0.05*
Non-farm wage vs own farm	-0.03*	-0.01	0.01	-0.01	0.01	-0.03
Non-farm salary vs own farm	-0.03*	0	-0.02	-0.02*	-0.01	-0.03
Non-farm business vs own farm	0	0	0	-0.01	0.05**	0.02
Other income vs own farm	-0.02	0	0.01	-0.01	0.04	-0.09**
Income quintile 2 vs income quintile 1	0.02	-0.02*	-0.03**	-0.01	0.02	-0.05**
Income quintile 3 vs income quintile 1	0.01	-0.04**	-0.02	-0.03**	0.03	0.01
Income quintile 4 vs income quintile 1	0	-0.01	-0.02*	-0.03**	0.10***	0.02
Income quintile 5 vs income quintile 1	-0.01	-0.02*	-0.01	-0.02**	0.09***	0.06**
Received remittances (0/1)	-0.02	-0.01	0.02*	0	0	0.05**
Battles/explosions/remote violence (#)	0	0	0	0	0.00**	-0.01
Feels insecure in community (0/1)	-0.02*	-0.03***	-0.02*	-0.03***	-0.03	0
Low level of social trust in community (0/1)	-0.01	-0.02	0	-0.02*	0.01	0.01
Climatic shock (0/1)	-0.02	-0.01	0	0.01	-0.08***	-0.12***
Altitude (mt)	0.00**	0	0.00***	0.00***	0.00***	0
Remote (0/1)	0.03***	0.03***	0.01	0.03***	0.01	0
Rural (0/1)	-0.02	0	-0.02	-0.02**	0.02	-0.05**
No. of Obs.	11514	11748	11881	23942	4714	3806

[a] Improved house refers to houses made from semi-pucca, bungalow/ brick, apartment/ condominium.
[b] Electricity connection includes households connected by national grid/private company/generator
Notes: Stars denote significant differences at p-values * $p < 0.10$, ** $p < 0.05$, *** $p < 0.01$.
Source: Authors' estimates from 2022 MHWS

5. Conclusion

COVID-19 led to an increase in socioeconomic phone surveys, but only a few of these aimed for and claimed national representativeness – let alone subnational level representativeness. A healthy dose of caution is indeed warranted in assuming that "national" phone surveys are representative (Brubaker et al. 2021). Phone ownership is not universal, and some respondents may be more able and willing to respond to a phone survey than others, thus potentially leading to over- or underrepresentation of certain groups among phone survey respondents. National in-person household surveys, however, also often require replacement of randomly selected enumeration areas with a nearby area due to inaccessibility for safety or other reasons. In some fragile states, such as Myanmar, entire geographical areas have been avoided by survey teams, thus leading to a non-negligible part of the population not being enumerated at all.

In this paper, we described a relatively novel approach to implementing a nationally and sub nationally representative phone survey from scratch, rather than from a pre-existing survey. The ingredients in this approach were:

1. A large and geographically dispersed database of phone numbers, in this case independently generated by the collaborating survey firm;
2. A target-based sampling strategy designed to reduce common phone survey biases (such as geographical bias, over-sampling of more educated and urban respondents) and to achieve gender parity as well as an over-sampling of sub-samples of interest (in this case, farm households); and
3. A multi-step construction of survey weights designed to further improve national and subnational representativeness.

Overall, the approach outlined in this study appears to be remarkably successful in generating a new nationally and regionally representative phone survey dataset. In terms of geographical coverage, the target system has proven relatively effective in reducing bias towards respondents from more geographically accessible locations or towards urban-based respondents. Indeed, our phone survey covers more townships than any previous nationally representative survey. Whereas past in-person survey teams had to avoid entire geographical areas affected by conflict, our phone survey sample does not contain such geographic bias.

The dataset also performs well in terms of estimating key demographic and wealth indicators. Weighted statistics for key variables that are roughly time-invariant closely match findings from other recent nationally representative surveys, including Individual-level respondent characteristics. It therefore seems to perform better than other phone survey datasets that have been thoroughly vetted (e.g. Maffioli 2020, Brubaker et al. 2021, Gourlay et al. 2021). Nevertheless, older people are underrepresented in our sample – both prior to and after weighting - compared to the age distribution of adults in the regular population, likely because of lower phone ownership among this group.

Time- and cost-considerations are also worth noting here. The survey was designed and implemented in a very short time span (a few months) and for much lower cost than in-person surveys (approximately one quarter of the cost of an in-person survey, though admittedly also for a

much shorter survey instrument). We estimate that our survey was also much more cost-effective compared to other phone surveys. For example, random digit dialing in step (1) – instead of a phone database with geographical location already known – would require a very large number of phone numbers to be called in order to achieve the targets outlined in step (2); obtaining sufficient numbers of households in small states/regions could be prohibitively costly. Indeed, we roughly estimate that random digit dialing is around twice as expensive as the approach used here. Moreover, these time and cost advantages offer additional advantages: phone surveys can be implemented at higher frequency, MHWS was implemented four times in 2022. This is particularly advantageous in volatile economic and political settings.

Despite a seemingly good performance of the sample – geographically and in terms of population characteristics – our assessment is limited to the available indicators comparable between our survey and other benchmark surveys. It is likely that there is some residual bias in certain respondent characteristics that we could not measure or control for. Geographically, even if at the township-level we have an adequate distribution, it is likely that residents of more remote locations or of acutely conflict-affected areas within each of these are not or less reached. In terms of demographic and wealth characteristics, we likely underrepresent respondents who do not easily communicate in Burmese or one of the other major ethnic languages and those at the highest and lowest percentage of the wealth distribution – the latter are likely non-phone owners. Many of these concerns, however, also pertain to in-person survey efforts.

Phone surveys also have other limitations. Phone surveys are short-duration interviews with a limited number of questions relative to in-person interviews. There is also some evidence that responses in phone surveys can be systematically different to those of in-person surveys (Lamanna et al. 2019), and that response fatigue may be at least as problematic in shorter phone surveys as it is in longer in-person surveys (Abay et al. 2021) and would also occur in repeated surveys (Schundeln 2018). That said, more research is needed to assess whether these are widespread problems or particular to the studied populations, survey modules, and modalities.

Bearing these caveats in mind, the potentially greater success of phone surveys in reaching areas affected by natural or human-made shocks, and their greater time- and cost-effectiveness for welfare monitoring, suggests that they should not be considered a mere “second-best” to in-person

survey efforts. We demonstrated an effective method for designing and implementing high-frequency phone surveys that are nationally and sub nationally representative. Collecting nationally and sub nationally representative data on key welfare indicators is critically important in fragile states such as Myanmar, where reliable data and rigorous research are increasingly scarce, yet also vitally important for targeting more resources to a growing population of vulnerable people.

Phone-based welfare surveillance systems have obvious advantages in conflict-affected states but monitoring individual and household welfare on a more frequent basis is important in almost any lower and middle income country context. Previous research already advocated for high frequency surveys focusing on the importance of climate change and other ecological and economic transformations, as well as seasonal shocks (Barret and Headey 2014; Headey and Barrett 2015). Agricultural economies are volatile at the best of times, but even urban economies in less developed countries are clearly highly vulnerable to the threats of further pandemics (GPMB 2019) and are affected by more frequent severe weather events induced by climate change (Seneviratne, et al. 2021). High frequency phone surveys can gauge many of the key impacts of these shocks and identify vulnerable households, the effectiveness of their coping mechanisms as well as external interventions, and potentially identify key trends–especially in agriculture–to inform early warning systems.

References

Abay KA, Berhane G, Hoddinott J, Hirfort KT. Assessing response fatigue in phone surveys: Experimental evidence on dietary diversity in Ethiopia. World Bank Policy Research Working Paper No. 2021.9636. The World Bank: Washington DC.

Barrett CB, Headey D. Measuring resilience in a volatile world: A proposal for a multicountry system of sentinel sites. Addis Ababa, Ethiopia. http://www.ifpri.org/publication/measuring-resilience-volatile-world. 2014.

Brubaker J, Kilic T, Wollburg P. Representativeness of individual-level data in COVID-19 phone surveys: Findings from Sub-Saharan Africa. PLOS ONE. 2021;16(11):e0258877.

CSO (Central Statistical Office), UNDP (United Nations Development Programme), WB (The World Bank). Myanmar Living Conditions Survey 2017: Poverty report. Yangon, Myanmar. 2019a.

CSO (Central Statistical Office), UNDP (United Nations Development Programme), WB (The World Bank). Myanmar Living Conditions Survey 2017: Technical report. Yangon, Myanmar. 2019b.

Dabalen A, Etang A, Hoogeveen J, Mushi E, Schipper Y, von Engelhardt J. Mobile phone panel surveys in developing countries: a practical guide for microdata collection. Directions in Development. Washington, D.C.: World Bank; 2016.

Demombynes G, Gubbins P, Romeo A. Challenges and Opportunities of Mobile Phone-Based Data Collection: Evidence from South Sudan. Policy Research Working Paper 6321. World Bank, Washington, DC; 2013.

Dillon B. Using mobile phones to collect panel data in developing countries. Journal of International Development. 2012;42(4):518-527.

DoP (Department of Population at the Ministry of Labour, Immigration, and Population). The 2014 Myanmar Population and Housing Census. The Union Report. Census Report Volume 2. Nay Pyi Taw, Myanmar; 2015.

DoP (Department of Population at the Ministry of Labour, Immigration, and Population). Mapping Myanmar's Nutrition in 2015. A Small Area Estimation. Nay Pyi Taw, Myanmar; 2020.

DoP (Department of Population at the Ministry of Labour, Immigration, and Population), UNFPA (United Nations Population Fund). The 2019 Inter-Censal Survey: Key findings, December 2020; 2020.

Etang A, Himelein K. Monitoring the Ebola Crisis Using Mobile Phone Surveys. In: Hoogeveen J, Pape U, editors. Data Collection in Fragile States: Innovations from Africa and Beyond. Cham: Springer International Publishing; 2020. p. 15-31.

Global Preparedness Monitoring Board (GPMB). A world at risk: annual report on global preparedness for health emergencies. Geneva: World Health Organization; 2019. https://www.gpmb.org/annual-reports/annual-report-2019

Gourlay S, Kilic T, Martuscelli A, Wollburg P, Zezza A. Viewpoint: High-frequency phone surveys on COVID-19: Good practices, open questions. Food Policy. 2021;105:102153.

Hainmueller J. Entropy balancing for causal effects: A multivariate reweighting method to produce balanced samples in observational studies. Political analysis. 2012;20(1):25-46.

Headey D, Barrett CB. Opinion: Measuring development resilience in the world's poorest countries. Proceedings of the National Academy of Sciences. 2015;112:11423-11425.

Henderson S, Rosenbaum M. Remote Surveying in a Pandemic: Research Synthesis. Innovation for Poverty Action; 2020.

Himelein K, McPherson L. Improved targeting for mobile phone surveys: A public-private data collaboration. https://blogs.worldbank.org/impactevaluations/improved-targeting-mobilephone-surveys-public-private-data-collaboration-guest. Accessed 04/01/2022. 2021.

Lamanna C, Hachhethu K, Chesterman S, Singhal G, Mwongela B, Ng'endo M, Passeri S, Farhikhtah A, Kadiyala S, Bauer J-M, Rosenstock TS. Strengths and limitations of computer assisted telephone interviews (CATI) for nutrition data collection in rural Kenya. PLOS ONE. 2019;14:e0210050.

Lau CQ, Cronberg A, Marks L, Amaya A. In Search of the Optimal Mode for Mobile Phone Surveys in Developing Countries. A Comparison of IVR, SMS and CATI in Nigeria. Survey Research Methods. 2019;13(3):305-318.

L'Engle K, Sefa E, Adimazoya EA, Yartey E, Lenzi R, Tarpo C, Heward-Mills NL, Lew K, Ampeh Y. Survey research with a random digit dial national mobile phone sample in Ghana: Methods and sample quality. PLOS ONE. 2018;13(1):e0190902.

Maffioli EM. Collecting Data During an Epidemic: A Novel Mobile Phone Research Method. Journal of International Development. 2020;32:1231-1255.

Schundeln M. Multiple Visits and Data Quality in Household Surveys. Oxford Bulletin of Economics and Statistics. 2018;80(2):380-405.

Seneviratne SI, Zhang X, Adnan M, Badi W, Dereczynski C, Di Luca A, Ghosh S, Iskandar I, Kossin J, Lewis S, Otto F, Pinto I, Satoh M, Vicente-Serrano SM, Wehner M, Zhou B. Weather and Climate Extreme Events in a Changing Climate. In: Climate Change 2021: The Physical Science Basis. Contribution of Working Group I to the Sixth Assessment Report of the Intergovernmental Panel on Climate Change [Masson-Delmotte V, Zhai P, Pirani A, Connors SL, Péan C, Berger S, Caud N, Chen Y, Goldfarb L, Gomis MI, Huang M, Leitzell K, Lonnoy E, Matthews JBR, Maycock TK, Waterfield T, Yelekçi O, Yu R, Zhou B (eds.)]. Cambridge University Press. In Press.

The World Bank. Weighting in high-frequency phone survey. Accessed online [https://microdata.worldbank.org/index.php/catalog/4035/download/50673]. 2021.

Wittenberg M. An introduction to maximum entropy and minimum cross-entropy estimation using Stata. The Stata Journal. 2010;10(3):315-330.

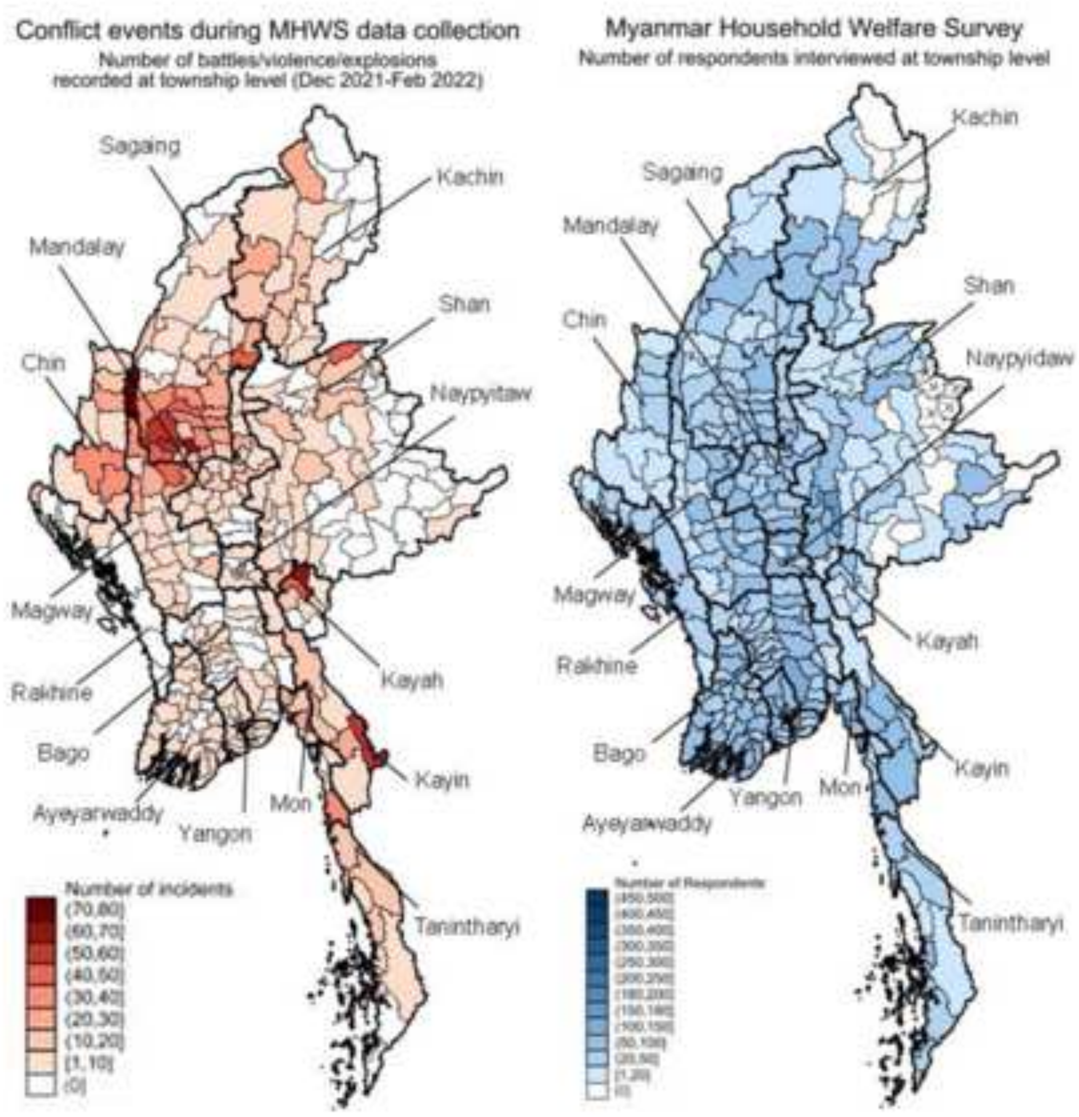
Conflict events during MHWS data collection
Number of battles/violence/explosions
recorded at township level (Dec 2021-Feb 2022)
Sagaing
Kachin
Mandalay
Shan
Chin
Naypyitaw
Magway
Rakhine
Kayah
Bago
Kayin
Ayeyarwaddy
Mon
Yangon
Tanintharyi
Number of incidents
(70,80]
(60,70]
(50,60]
(40,50]
(30,40]
(20,30]
(10,20]
[1, 10]
{0}
Myanmar Household Welfare Survey
Number of respondents interviewed at township level
Kachin
Sagaing
Mandalay
Shan
Chin
Naypyidaw
Magway
Kayah
Rakhine
Bago
Kayin
Yangon
Mon
Ayeyawaddy
Tanintharyi
Number of Respondents

Figure

Click here to access/download;Figure;S1fig.tif

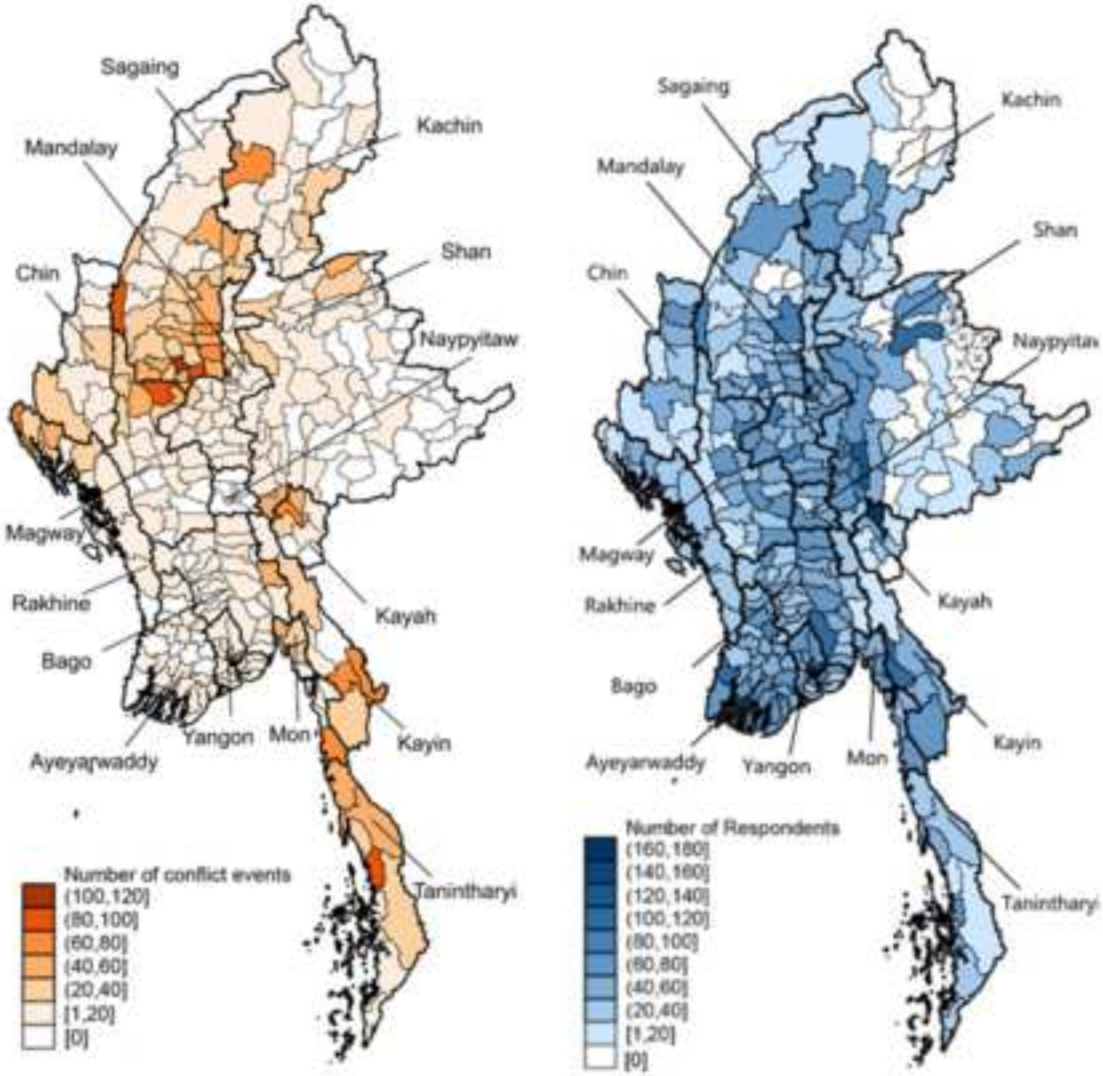

Figure Click here to access/download;Figure;S2fig.tif

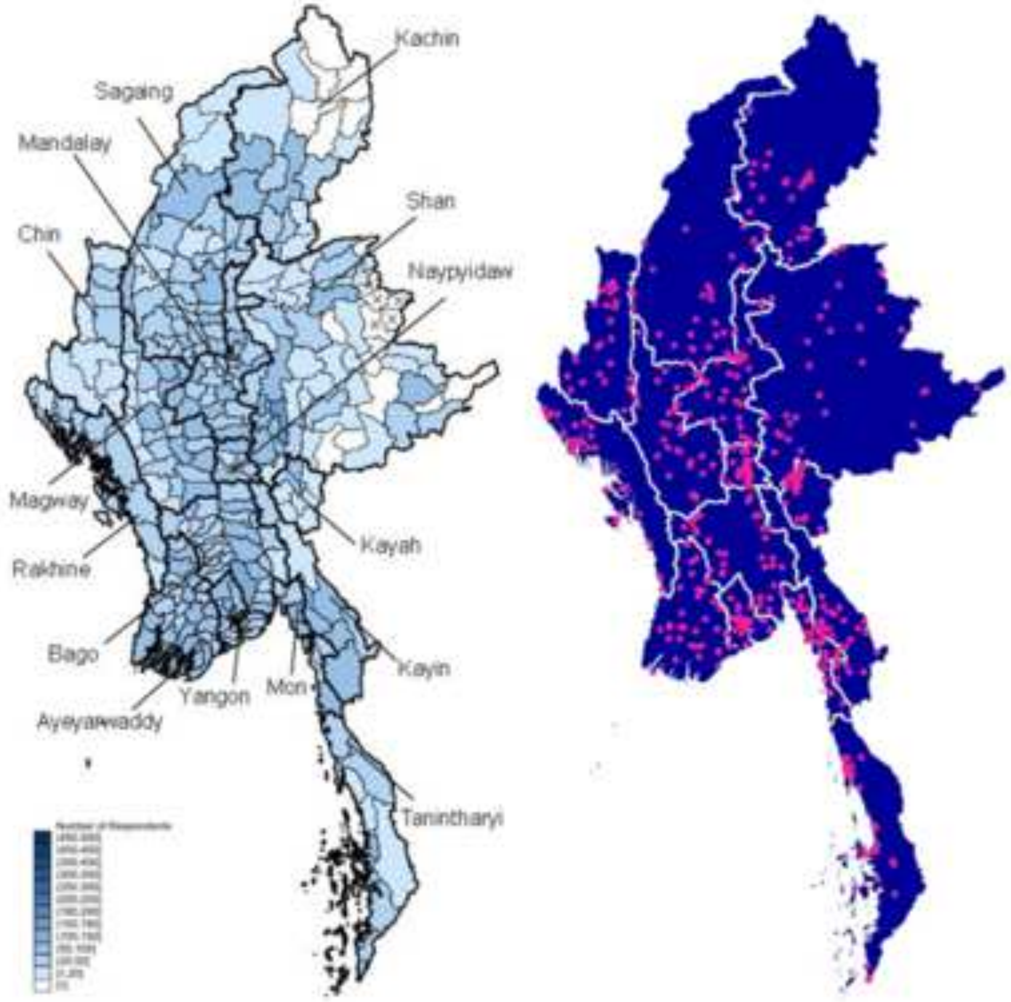

Figure
Click here to access/download;Figure;S3fig.tif

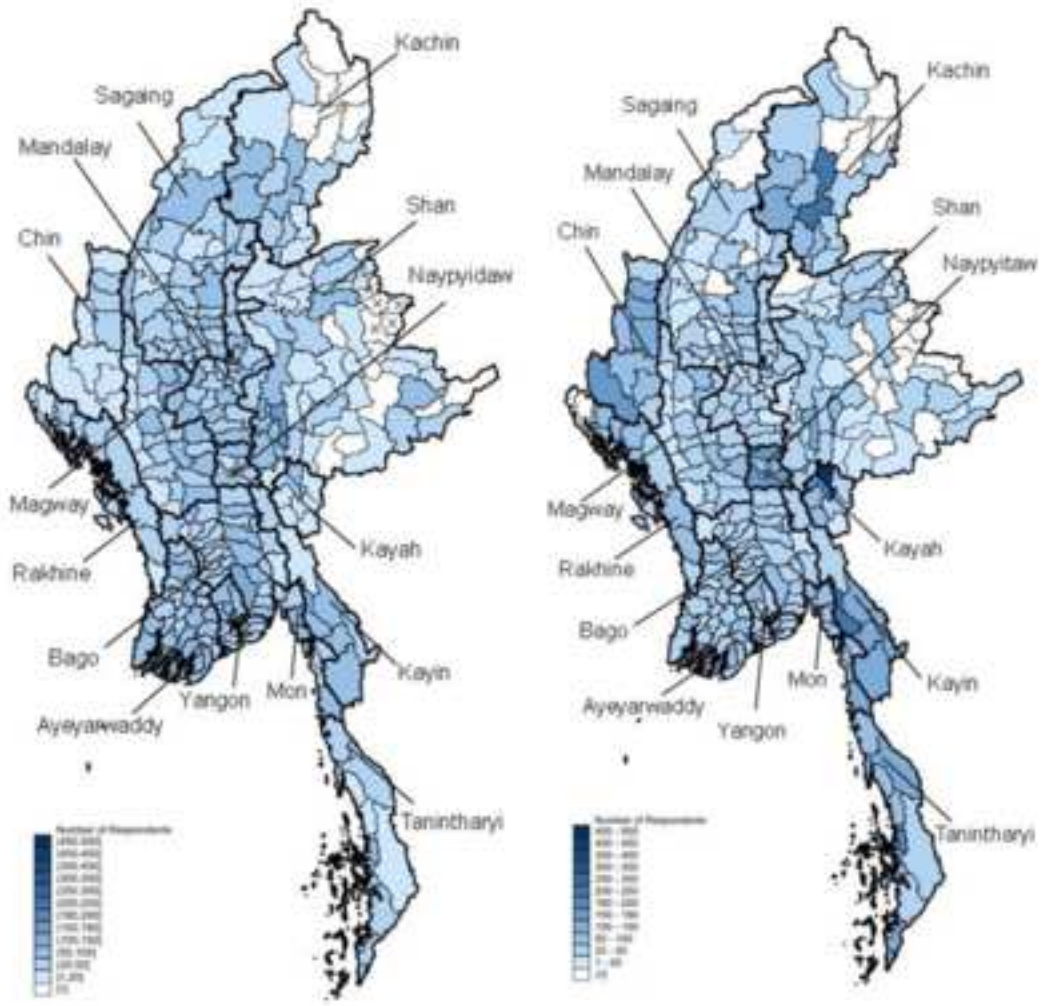

Click here to access/download
Supporting Information
Supporting information.docx
