# Supplementary material for: Can phone surveys be representative in low- and middle-income countries? An application to Myanmar
Source: PLoS One. 2023 Dec 22;18(12):e0296292. doi: 10.1371/journal.pone.0296292 (PMC10745169; doi:10.1371/journal.pone.0296292)
Supplement: Attachment — Submitted filename: Respose to Reviewers.docx [file pone.0296292.s005.docx]

December 1, 2023

Ref: Resubmission of Manuscript PONE-D-23-25141

Dear Editor:

We appreciate the opportunity to revise our research manuscript " Can phone surveys be representative in low- and middle-income countries? An application to Myanmar.” We revised the document according to your recommendations and the reviewers’ thoughtful comments. Below we explain in detail how we have handled the specific comments. For clarity, we first reproduce the original comments, in bold italics, followed by our response, in roman font, and the new lines in the text, in italics.

Reviewer 1

***Thank you for your thorough responses to my 47 (!!) comments.
Along with the feedback and updates suggested by Reviewer 3, the manuscript is much improved.***

***The remaining piece is to improve the discussion section.
Two comments on this:

1- in the results section, you added new information about attrition. it is a great add but you have discussion section information there - starting with "Whether our phone survey out- or ... but attirition rates among respondents in the other regions were low (1 percent of hte remaining sample) [28]." belongs in discussion section***

Thank you very much for your commitment to improving the quality of our paper. We have made the following changes in response to your comments. We have added a discussion section and now placed the discussion on attrition in the discussion section as part of the discussion of studies that are consistent or contrast with our study.

***2- discussion section should be shaped as follows:

·  Summarize your findings (Give the key results that have lessonslearning for the others to replicate.)· 
 Studies that are consistent or in contrastwith your study· 
 What your study adds·  
Strengths and limitations
·  Conclusion***

We have added a discussion section and followed the format that you have recommend. First, we highlight our findings and the key results for other authors to replicate (page 23 paragraph 1). Next, we highlight other studies that are consistent or in contrast with our study and emphasize what our study adds to the field on pages 24 and 25. This is followed by a discussion of strengths and weaknesses on page 27. We hope this new format will make the discussion clearer and more thorough.

Reviewer 3

***I commend the authors for thoroughly addressing my comments. I am recommending to accept this paper.***

Thank you very much for your thorough review of our paper, and continued commitment to improving the quality of our interpretation of the literature.

***I have two minor follow-up comments for the authors, which however do not require a formal revision. If the authors see fit they can incorporate these comments.***

***1-First, on attrition. I believe the Gourlay et al. paper has some information on attrition for the series of surveys they cover, which may be worth referencing.***

Thank you for pointing this out, we have now included the following lines in our discussion section.

*When comparing the response rate to the COVID-19 high frequency surveys implemented among participants in recent in-person Living Standard Measurement Surveys (LSMS) in five different countries in Sub-Saharan Africa, as many as 94 percent of households with phone numbers were reached in Uganda but only 62 percent Ethiopia [10].*

***2-Second, on weighting. I share the general conclusion of the authors that weighting can only do so much to improve representativeness and it is also fair to say that weighting has somewhat mixed results especially if initial differences are large. However, the Brubaker et al paper does not entirely support this conclusion. I went back to the paper, and my read is that the authors are referring to the fact that household weights (w1) can make things worse, while individual weights (w2) at least do not make things worse. This is my read of this figure:***

We are sorry for overlooking this, we now understand that Brubaker et al. improve their individual-level estimates if they use the individual-level weights, which are propensity score-adjusted individual weights. We improved our text on page 19 to highlight this point.

*In contrast, such approach was not satisfactory for individual representativeness in several COVID-19 phone surveys in Sub-Saharan Africa [7], and further recalibration of phone survey sampling weights with propensity score adjustments (based on the individual’s likelihood to be interviewed) was necessary to ensure that their individual-level characteristics would be closer to those of the benchmark population.*
